# Genome-wide identification of the mitogen-activated kinase gene family from *Limonium bicolor* and functional characterization of LbMAPK2 under salt stress

**DOI:** 10.1186/s12870-023-04589-x

**Published:** 2023-11-15

**Authors:** Caixia Zhang, Zhihui Zhu, Aijuan Jiang, Qing Liu, Min Chen

**Affiliations:** 1 Shandong Provincial Key Laboratory of Plant Stress Research, College of Life Sciences, Shandong Normal University, Shandong, 250014 China; 2 Dongying Institute, Shandong Normal University, No. 2 Kangyang Road, Dongying, Shandong 257000 China

**Keywords:** Mitogen-activated kinase, *Limonium bicolor *(Bunge) Kuntze, Salt tolerance

## Abstract

**Background:**

Mitogen-activated protein kinases (MAPKs) are ubiquitous signal transduction components in eukaryotes. In plants, MAPKs play an essential role in growth and development, phytohormone regulation, and abiotic stress responses. The typical recretohalophyte *Limonium bicolor *(Bunge) Kuntze has multicellular salt glands on its stems and leaves; these glands secrete excess salt ions from its cells to mitigate salt damage. The number, type, and biological function of *L. bicolor MAPK* genes are unknown.

**Results:**

We identified 20 candidate *L. bicolor MAPK* genes, which can be divided into four groups. Of these 20 genes, 17 were anchored to 7 chromosomes, while *LbMAPK18*, *LbMAPK19*, and *LbMAPK20* mapped to distinct scaffolds. Structure analysis showed that the predicted protein LbMAPK19 contains the special structural motif TNY in its activation loop, whereas the other LbMAPK members harbor the conserved TEY or TDY motif. The promoters of most *LbMAPK* genes carry *cis*-acting elements related to growth and development, phytohormones, and abiotic stress. *LbMAPK1*, *LbMAPK2*, *LbMAPK16*, and *LbMAPK20* are highly expressed in the early stages of salt gland development, whereas *LbMAPK4*, *LbMAPK5*, *LbMAPK6*, *LbMAPK7*, *LbMAPK11*, *LbMAPK14*, and *LbMAPK15* are highly expressed during the late stages. These 20 LbMAPK genes all responded to salt, drought and ABA stress. We explored the function of LbMAPK2 via virus-induced gene silencing: knocking down *LbMAPK2* transcript levels in *L. bicolor* resulted in fewer salt glands, lower salt secretion ability from leaves, and decreased salt tolerance. The expression of several genes [*LbTTG1* (*TRANSPARENT TESTA OF GL1*), *LbCPC* (*CAPRICE*), and *LbGL2* (*GLABRA2*)] related to salt gland development was significantly upregulated in *LbMAPK2* knockdown lines, while the expression of *LbEGL3* (*ENHANCER OF GL3*) was significantly downregulated.

**Conclusion:**

These findings increase our understanding of the *LbMAPK* gene family and will be useful for in-depth studies of the molecular mechanisms behind salt gland development and salt secretion in *L. bicolor*. In addition, our analysis lays the foundation for exploring the biological functions of MAPKs in an extreme halophyte.

**Supplementary Information:**

The online version contains supplementary material available at 10.1186/s12870-023-04589-x.

## Background

Signaling cascades based on mitogen-activated protein kinases (MAPKs) are ubiquitous and participate in signal transduction in all eukaryotes [1]. Each MAPK signal transduction module is typically composed of three kinases. Generally, the most upstream MAPK kinase kinase (MAPKKK) is activated after perceiving external signals, after which the MAPKKK phosphorylates a downstream MAPK kinase (MAPKK); the phosphorylated MAPKK ultimately phosphorylates its downstream MAPK [2]. MAPKs are activated when both threonine (T) and tyrosine (Y) residues in the TXY motif [with “X” representing Aspartic acid (D) and Glutamic acid (E) residues] within their activation loops are phosphorylated by their upstream MAPKKs [2–4]. Phosphorylated MAPKs go on to phosphorylate other signaling molecules [5].

In a MAPK cascade, each level is encoded by a small gene family, and multiple members can function redundantly [6]. The members of the *MAPK* gene family function downstream of a specific receptor and coordinate cells to achieve the normal growth and development of organisms, as well as adjusting to changing environments. MAPKs are a complex protein family in plants with many members [7–9]. For example, the *MAPK* gene family of Arabidopsis (*Arabidopsis thaliana*) consists of 20 members [5]. The maize (*Zea mays*) genome contains at least 19 *MAPK* genes [10], and 16 *MAPK* genes have been reported in the Tartary buckwheat (*Fagopyrum tataricum*) genome [11].

The MAPK family plays important roles in plant growth and development and the response to abiotic stresses [12]. For instance, Asif et al. [13] used a genome-wide identification and expression analysis of the entire gene family of banana (*Musa* sp.) to show that MaMPKs may be involved in the ethylene signal transduction pathway of fruit ripening. In apple (*Malus domestica*), MdMPK4 promotes anthocyanin accumulation by phosphorylating the MYB-type transcription factor MdMYB1 [14]. Great progress has been made in studying MAPKs and their roles in stress signaling cascades [15]. Indeed, GmMMK1, a model of salt-related MAPKs, plays important roles in the salt stress response of soybean (*Glycine max*) seedlings, negatively regulating the salt stress response. Overexpressing *GmMMK1* in soybean hairy roots increases the sensitivity of these *GmMMK1*-OE plants to salt stress [16]. In rice (*Oryza sativa*), overexpressing *OsMAPK33* decreases tolerance to salt stress [17]. Under salt stress, FtMAPK1 can improve the stress tolerance of Tartary buckwheat by upregulating the expression of stress-related genes and raising the activity of antioxidant enzymes [11]. Therefore, MAPKs are not only involved in various aspects of plant physiology but also in plant responses to abiotic stresses.

As a typical recretohalophyte, the sea lavender *Limonium bicolor* secretes excess salt ions from its body to the outside milieu through specialized salt glands present on the surface of its leaves and stems to mitigate the harm imposed by high intracellular salt concentrations [18, 19]. In fact, the salt tolerance of *L. bicolor* depends mainly on the density of salt glands on its leaves and the salt excretion ability of its salt glands [20].

MAPKs play an important role in plant responses to salt stress, transmitting signals from the cell surface to the nucleus [14, 15]. Whereas the importance of MAPKs in plant growth and development and in response to stress has been described in other plant species [12, 13], little is known about the contribution of MAPKs to the salt-stress response in salt-secreting halophytes, especially the evolution and functional information of this gene family in *L. bicolor*. To address this knowledge gap, we identified 20 *LbMAPK*s in *L*. *bicolor* and analyzed their phylogeny, chromosomal locations, the *cis*-acting elements present in their promoter regions, the functional domains in their encoded proteins, and their function in the acclimation of *L*. *bicolor* to salt stress. On the basis of their expression levels, we show that the *LbMAPK* gene family is involved in responses to salt stress, osmotic stress, and abscisic acid (ABA) signaling, and positively regulates salt tolerance of *L*. *bicolor* by promoting salt gland development and salt secretion.

## Results

### Identification of *MAPK* gene family members from *L. bicolor* and their chromosomal distribution

**Fig. 1:**
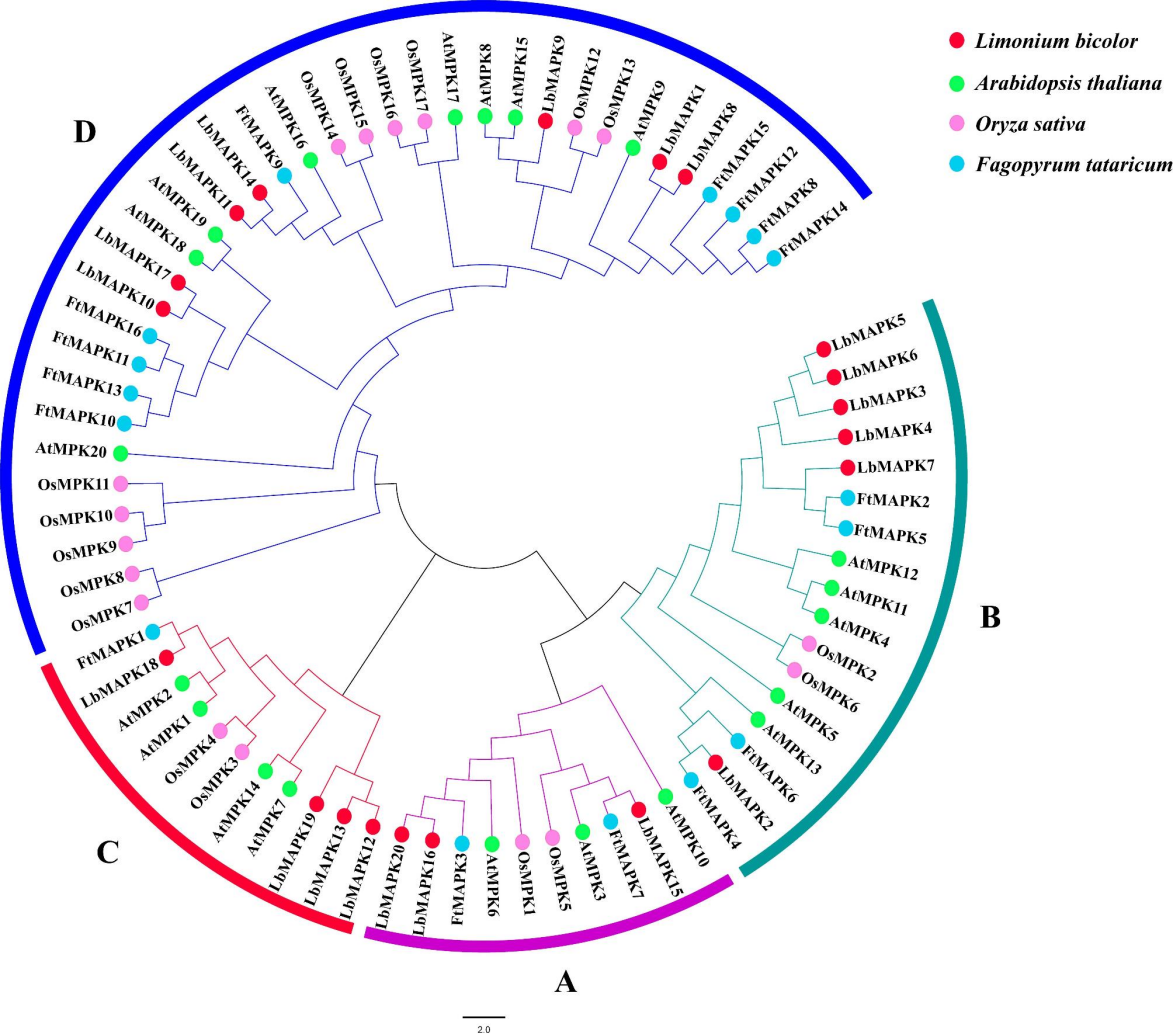
Phylogenetic relationship of putative MAPKs in *L. bicolor*, *F. tataricum*, *A. thaliana*, and *O. sativa*. The phylogenetic tree was created using the NJ (Neighbor-Joining) method. Bootstrap values were determined by 1000 replicates and are indicated on each branch. **A**, **B**, **C**, and **D** exhibit diferent gene clusters of MAPKs and four different colored dots represent different species

**Fig. 2:**
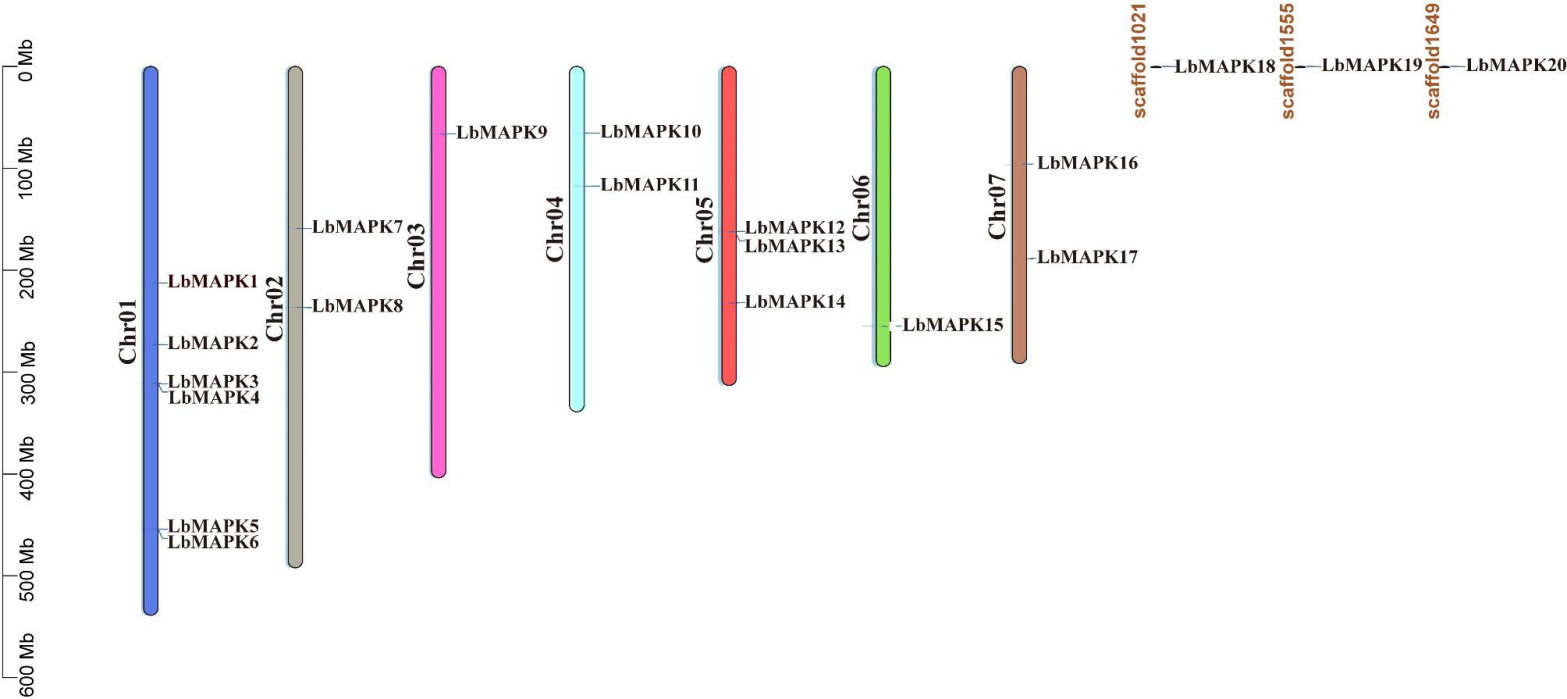
Schematic representations of the chromosomal distributions of the *LbMAPK* genes. The vertical bars in different colors mark the chromosomes of *L. bicolor*. The chromosome number is located to the left of each chromosome. The scale on the left represents the chromosome length

To analyze the evolutionary history and characterize the *LbMAPK* family, we identified 20 *MAPK* candidate genes in the recently published *L. bicolor* genomic sequence (https://www.ncbi.nlm.nih.gov/sra/?term=Limonium%20Bicolor%20); we designated these genes as *LbMAPK1* to *LbMAPK20* according to their position on the chromosomes (Figs. 1 and 2). We were able to assign 17 members (*LbMAPK1* to *LbMAPK17*) to one of the seven *L. bicolor* chromosomes, while *LbMAPK18*, *LbMAPK19*, and *LbMAPK20* only mapped to Scaffold1021, Scaffold1555, and Scaffold1649, respectively (Fig. 2). As illustrated in Fig. 2, one *LbMAPK* gene mapped to each of chromosomes 03 and 06, with two *LbMAPK* genes being located on chromosomes 02, 04, and 07, respectively. Almost half of all *LbMAPK* genes were located on chromosomes 01 (six genes) and 05 (three genes). A search for conserved functional domains in all predicted LbMAPK proteins revealed the conserved amino acid residues of the TXY motif located within the kinase activation loop of each one, with the threonine (T) and tyrosine (Y) being targets for phosphorylation by the upstream MAPKK (Figure S1). A Batch-CD Search (https://www.ncbi.nlm.nih.gov/Structure/bwrpsb/bwrpsb.cgi) and TBtools visualization analysis indicated that all LbMAPKs contain a TEY, TDY, or TNY phosphorylation motif.

We analyzed the characteristics of the 20 LbMAPKs. These proteins ranged in length from 240 amino acids (aa) with a molecular weight of 27.50 kDa to 776 aa and a molecular weight of 89.38 kDa. The isoelectric point (pI) varied from 4.94 (LbMAPK2) to 9.40 (LbMAPK17). Moreover, we predicted the subcellular localization of each LbMAPK: of the 20 proteins, nine were predicted to localize to the chloroplast and 10 to the cytoplasm, with only LbMAPK11 predicted to be located in mitochondria. All the *LbMAPK*s identified in this study, with their gene names, gene IDs, and other related information are provided in Table 1.

**Table 1:** Characteristics of 20 *MAPK* genes in *L. bicolor*

Gene name	Gene ID	Chr	Start	End	PL(aa)	MW(KDa)	PI	T loop	SL
LbMAPK1	Lb1G01452.1	Chr01	212,435,232	212,442,711	555	62.87	6.88	TDY	Chloroplast
LbMAPK2	Lb1G02559.1	Chr01	272,915,797	272,919,957	369	42.37	4.94	TEY	Cytoplasm
LbMAPK3	Lb1G03560.1	Chr01	310,910,402	310,911,566	240	27.50	5.49	TEY	Cytoplasm
LbMAPK4	Lb1G03562.1	Chr01	310,928,218	310,931,606	285	32.58	6.72	TEY	Chloroplast
LbMAPK5	Lb1G07412.1	Chr01	454,136,931	454,141,003	379	43.48	6.36	TEY	Cytoplasm
LbMAPK6	Lb1G07414.2	Chr01	454,150,247	454,154,225	378	43.50	6.50	TEY	Cytoplasm
LbMAPK7	Lb2G11155.1	Chr02	158,816,995	158,819,914	385	44.01	6.55	TEY	Cytoplasm
LbMAPK8	Lb2G12330.2	Chr02	236,457,801	236,464,308	554	62.97	6.41	TDY	Chloroplast
LbMAPK9	Lb3G16537.1	Chr03	66,305,306	66,310,028	605	68.63	6.39	TDY	Chloroplast
LbMAPK10	Lb4G23192.1	Chr04	65,389,296	65,394,926	632	71.88	9.32	TDY	Chloroplast
LbMAPK11	Lb4G24221.1	Chr04	117,627,168	117,630,883	526	59.52	8.73	TDY	Mitochondrion
LbMAPK12	Lb5G27787.1	Chr05	162,150,639	162,154,510	776	89.38	6.21	TDY	Cytoplasm
LbMAPK13	Lb5G27798.1	Chr05	162,528,420	162,530,441	528	60.77	5.85	TDY	Cytoplasm
LbMAPK14	Lb5G29170.1	Chr05	232,195,470	232,199,192	553	62.76	8.62	TDY	Chloroplast
LbMAPK15	Lb6G31696.1	Chr06	255,006,133	255,009,039	256	29.85	6.15	TEY	Cytoplasm
LbMAPK16	Lb7G33589.1	Chr07	95,969,660	95,975,045	395	45.41	5.63	TEY	Chloroplast
LbMAPK17	Lb7G34942.1	Chr07	188,453,407	188,459,173	627	71.14	9.40	TDY	Chloroplast
LbMAPK18	Lb0G36729.1	scaffold1021	7138	11,119	376	42.98	6.64	TEY	Cytoplasm
LbMAPK19	Lb0G37133.1	scaffold1555	3153	4570	405	46.65	6.77	TNY	Chloroplast
LbMAPK20	Lb0G37186.1	scaffold1649	3915	8348	279	32.75	5.26	TEY	Cytoplasm

Chr: chromosomal location, Start and End: start and end position of the genes on chromosome, PL: protein length, MW: molecular weight, PI: isoelectric points, SL: subcellular location, T loop: activation domain

### Phylogenetic relationships of LbMAPK proteins

To explore the evolutionary relationships of these putative LbMAPKs, we reconstructed a phylogenetic tree based on the protein sequences of the 20 LbMAPKs, 20 MAPKs from Arabidopsis, 17 OsMPKs from rice, and 16 FtMAPKs from Tartary buckwheat (Fig. 1). All MAPK proteins could be divided into four groups, designated A to D (Fig. 1). Each group contained MAPKs from all four plant species. Furthermore, we noticed that the distribution of LbMAPKs within each group was not uniform. Notably, LbMAPKs belonging to the same group in the phylogenetic tree were not restricted to the same chromosomes, but also existed on different chromosomes or along the length of the same chromosomes (Figs. 1 and 2).

We did, however, notice that LbMAPKs from groups A and B are characterized by the conserved phosphorylation motif TEY; LbMAPKs from group D contained the TDY motif, whereas LbMAPK members from group C contained the TDY (LbMAPK12 and LbMAPK13), TEY (LbMAPK18), or TNY (LbMAPK19) motif. The number of MAPKs from group D was comparable among three of the species: seven in *L. bicolor*, eight in Arabidopsis, and nine in Tartary buckwheat; only the rice genome encodes more group-D MAPKs, with 11 members (Fig. 1).

### Gene structure of *LbMAPKs*, conserved motifs and conserved domains of LbMAPKs

**Fig. 3:**
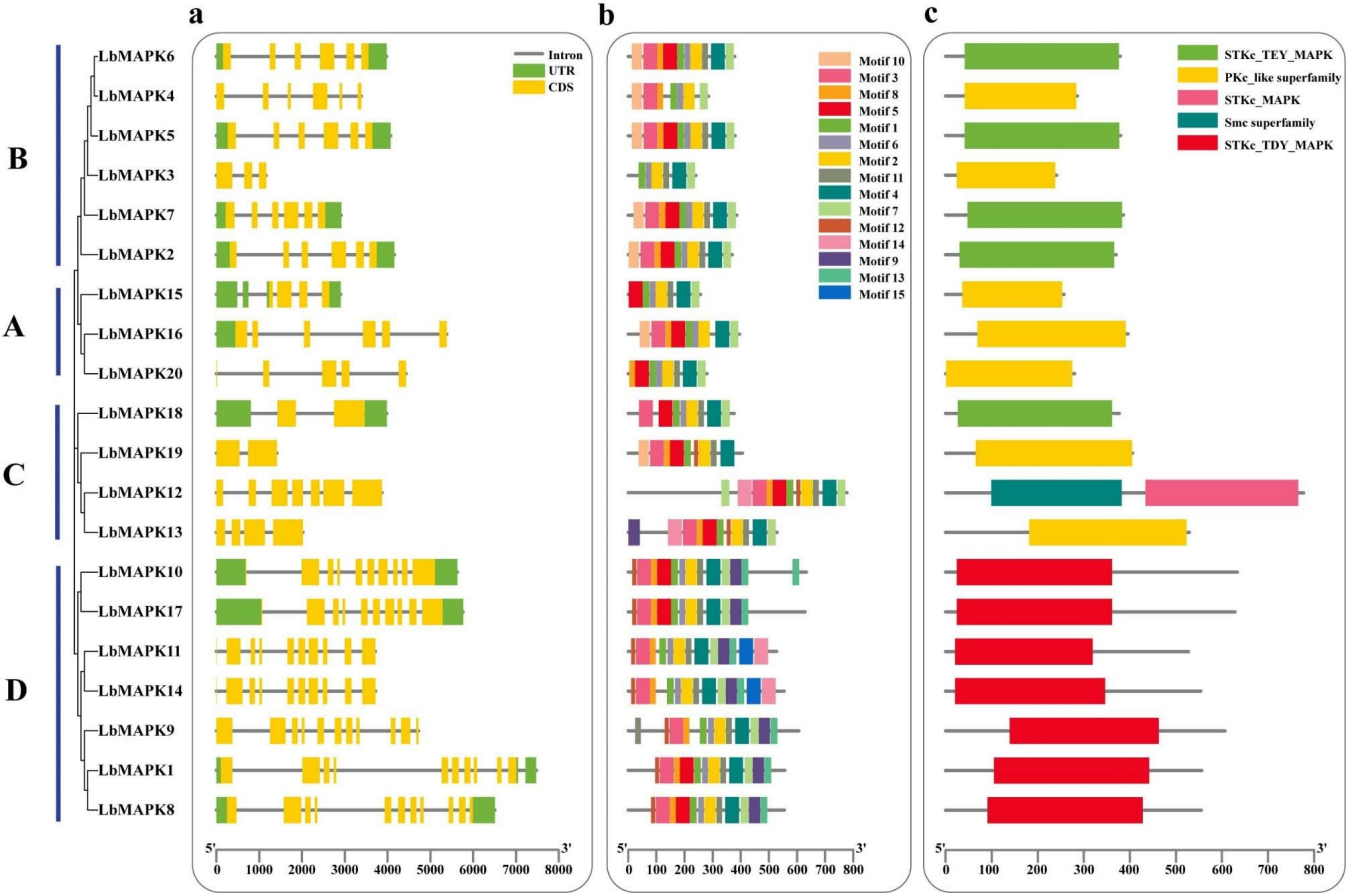
Characterization of LbMAPKs. **a** Motif composition of LbMAPK proteins. 15 conserved motifs in LbMAPK proteins are indicated by multiple colored boxes. **b** The exon and intron structures of *LbMAPK* genes. Yellow boxes represent exons gray lines represent introns. **c** Predicted domain of MAPK proteins in *L. bicolor.* The different colored rectangles represent different conserved domains

To validate the authenticity of the 20 putative *LbMAPK* genes identified in this study and explore the diversity of this gene family, we analyzed the structure of exons and introns of all 20 *LbMAPK* genes (Fig. 3a). We grouped the 20 *LbMAPK* genes according to their phylogenetic relationships in the above tree with adjustments based on the underlying gene structure. The number of exons in group-D *LbMAPK*s was greater than that for the other groups; in addition, the length of exons and introns changed greatly among group members. Indeed, the number of introns ranged from 1 to 10, and the number of exons from 2 to 11. Only in group D did most of the genes have a similar genomic structure. We also analyzed the LbMAPKs for conserved functional motifs and found some proteins containing up to 13 motifs. Notably, only motif 2 was present in all MAPK family members (Fig. 3b). Motifs 4 and 7 were present in all MAPKs except LbMAPK4 and LbMAPK19. In addition, motif 9 was common to all group-D MAPKs (Fig. 3).

### Analysis of *cis*-acting elements in *LbMAPK* promoters

**Fig. 4:**
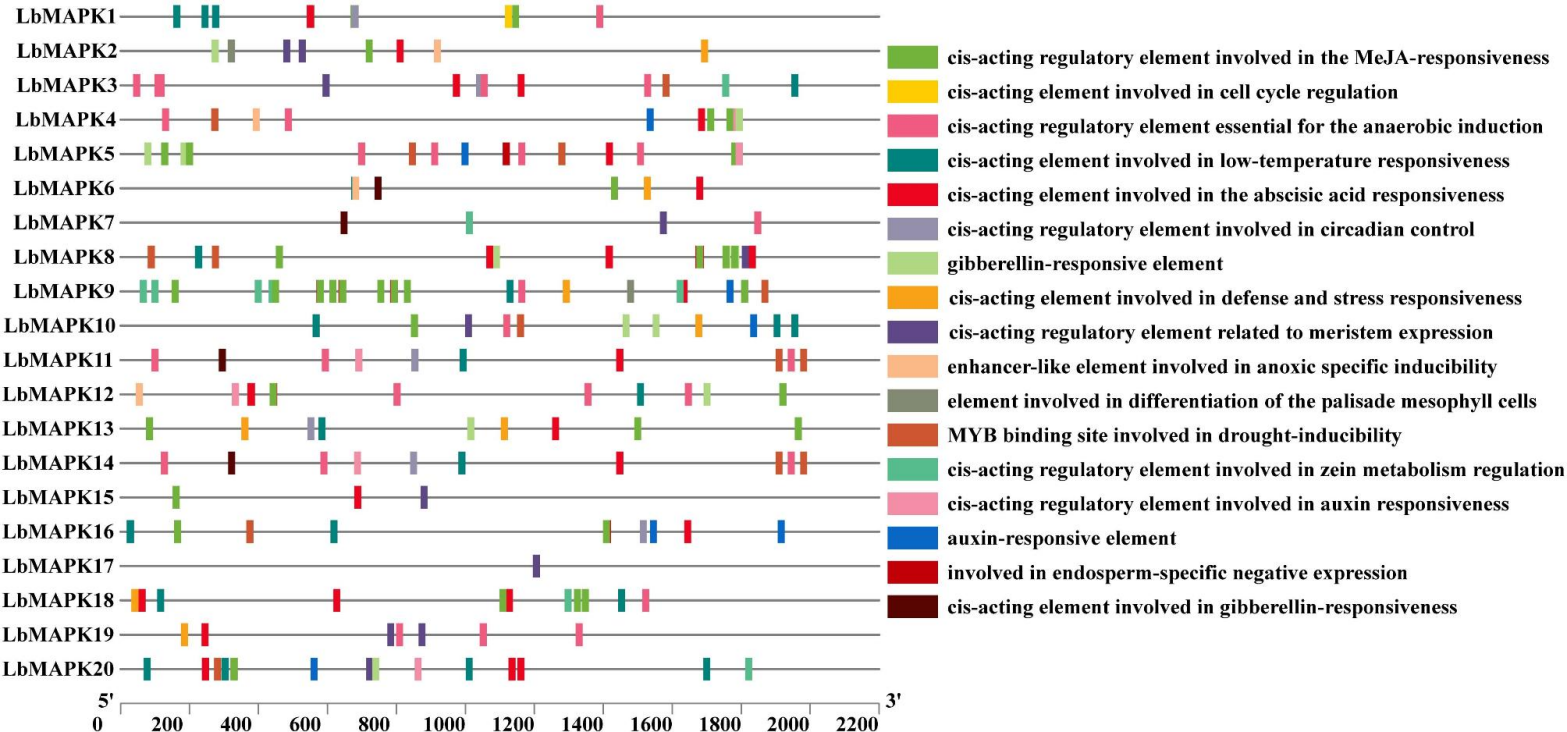
*Cis*-acting elements in the promoter regions of 20 *LbMAPKs*. *Cis*-elements with similar functions are displayed in the same colour. The black line indicates the promoter length of the *LbMAPK* genes. The different coloured boxes on the right represent *cis*-acting elements with different functions

To better understand the regulation of *LbMAPK*s during stress responses, we investigated the complement of *cis*-acting elements in the promoter regions of *LbMAPK* genes, using the 2.2-kb region upstream of each translation start codon (Fig. 4). This analysis suggested that the promoters of most *LbMAPK*s contain phytohormone [auxin, methyl jasmonate (MeJA), and ABA]-responsive elements. Notably, the *LbMAPK17* promoter only contained one *cis*-acting element, associated with meristem expression, while the *LbMAPK9* promoter contained the 21 *cis*-acting elements. We hypothesized that all *LbMAPK* genes are involved in abiotic stress responses, as suggested by *cis*-acting elements related to stress and defense responsiveness in the promoters of seven *LbMAPK*s (*LbMAPK2*, *LbMAPK6* and *LbMAPK9* etc.) and *cis*-regulatory elements involved in low-temperature responsiveness in the promoters of 13 *LbMAPK*s (*LbMAPK1*, *LbMAPK3* and *LbMAPK6* etc.). We also predicted that several *LbMAPK*s (*LbMAPK3*, *LbMAPK4* and *LbMAPK5* etc.) may be involved in drought responses in conjunction with a MYB transcription factor (R2R3-type MYB TF). Other *cis*-acting elements suggested the expression of *LbMAPK*s (*LbMAPK2*, *LbMAPK3* and *LbMAPK7* etc.) in meristems (Fig. 4). The *LbMAPK15* promoter contains three *cis*-acting elements that respond to MeJA, ABA, and drive meristem expression, respectively. These results underscore the potential function of LbMAPKs in plant growth and development, phytohormone regulation, and response to abiotic stresses.

### Expression of *LbMAPK*s during salt gland development

**Fig. 5:**
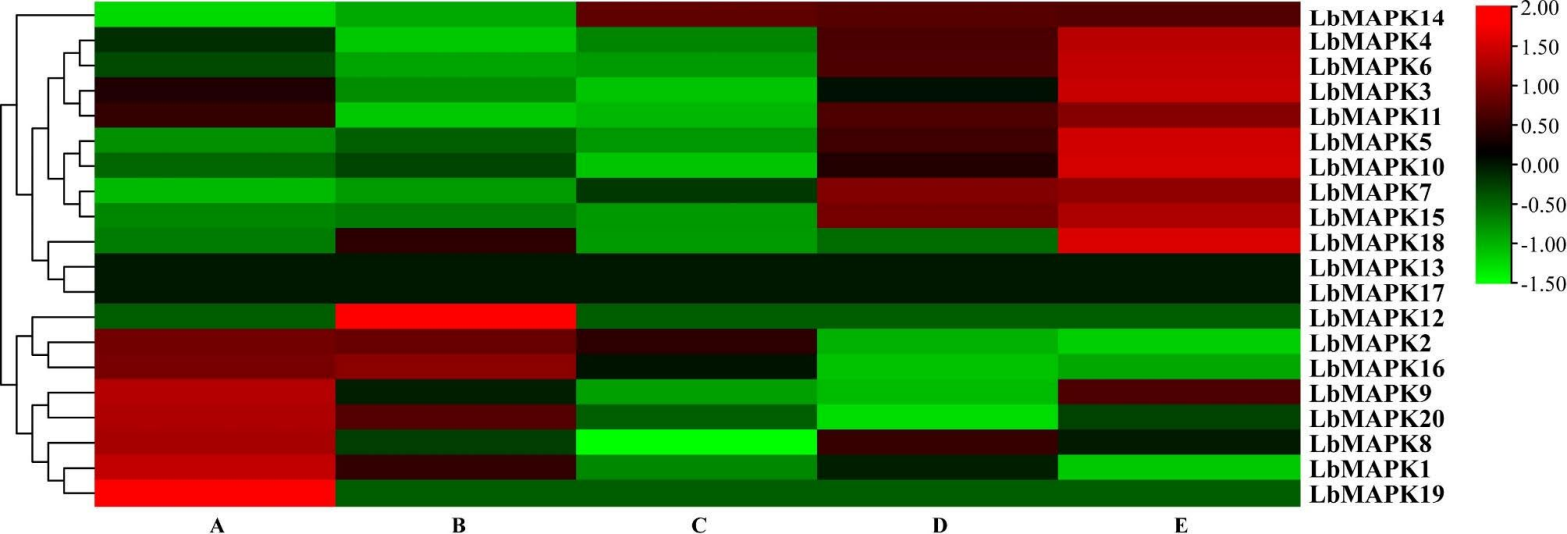
Expression analysis of *LbMAPKs* during salt gland development. Heat map showing expression profiles of *LbMAPKs* in different stages of salt gland development. Green color indicates low expression, black color indicates medium expression, red color indicates high expression

Analyzing the expression patterns of *MAPK* genes can offer important clues about their functions, including during salt gland development and in salt tolerance in *L. bicolor.* The development of salt glands can be divided into five stages: A (earliest) through E (latest) [21]. We explored the expression levels of *LbMAPK*s during salt gland development from a previously published transcriptome database of *L. bicolor* salt gland development [22]. As shown in Fig. 5, *LbMAPK1*, *LbMAPK2*, *LbMAPK16*, and *LbMAPK20* were highly expressed in the early stages of salt gland development (stages A and B), whereas *LbMAPK4*, *LbMAPK5*, *LbMAPK6*, *LbMAPK7*, *LbMAPK11*, *LbMAPK14*, and *LbMAPK15* were highly expressed in the later stages of salt gland development (stages D and E). *LbMAPK13* and *LbMAPK17* were hardly expressed at any stage of salt gland development, suggesting that these two genes are not involved in salt gland development.

### Expression of *LbMAPK*s under stress conditions

**Fig. 6:**
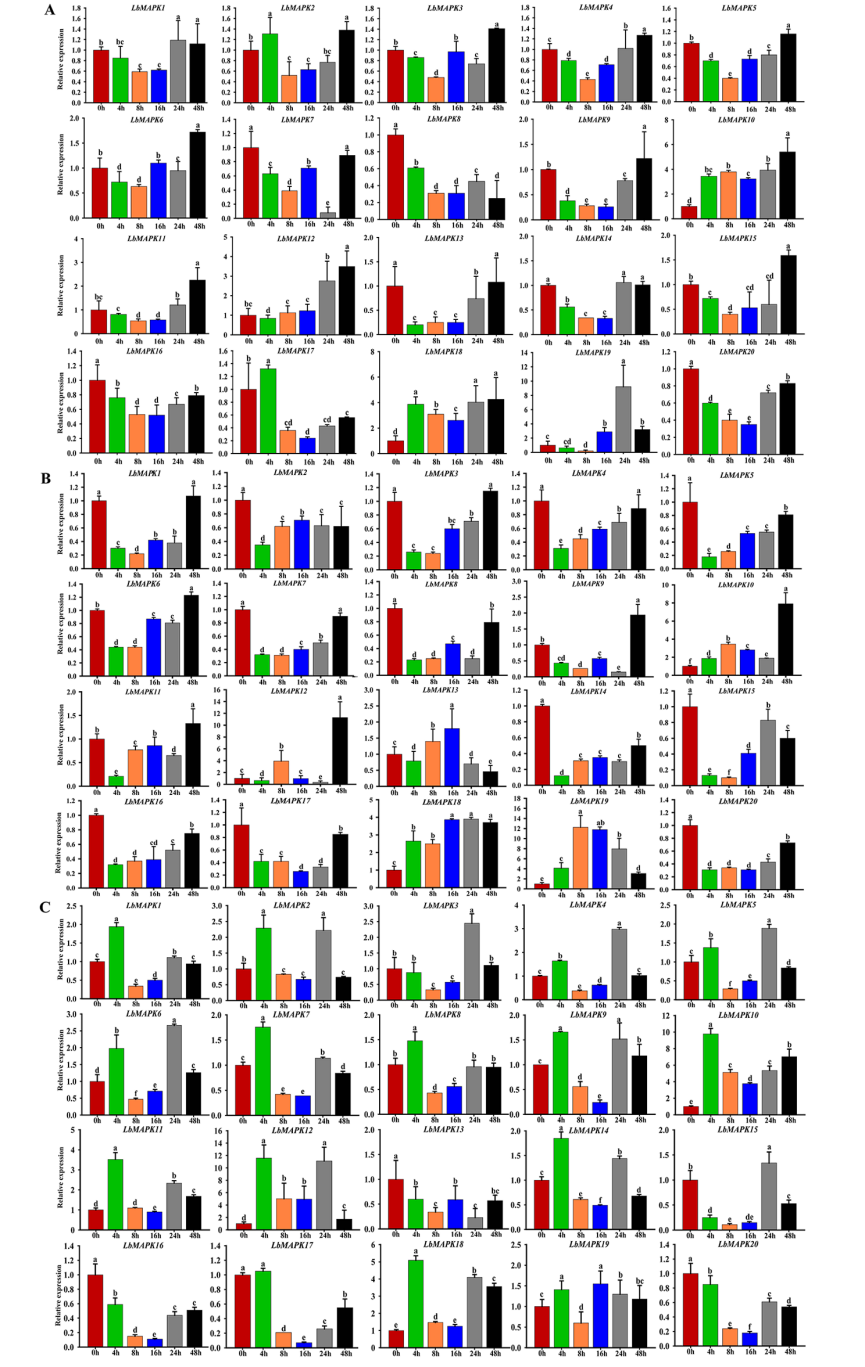
Expression analysis of *LbMAPKs* under stress conditions. **A**, **B** and **C** represent the relative expression of *LbMAPKs* in the leaves of *L. bicolor* treated with 100 μM ABA, 20% PEG and 200 mM NaCl for different time (0 ~ 48 h), respectively. The expression level of all genes in 0 h was set as 1. Three replicates should be set up to calculate the standard deviation (SD) and ensure accuracy. Letters above the bar (a-f) were used to indicate significant differences between different columns (*p* = 0.05, Duncan)

We explored the potential functions of LbMAPKs in the phytohormone response or during abiotic stress by investigating their expression patterns following treatment with 100 µM ABA, 20% (w/v) polyethylene glycol (PEG) to simulate drought stress, or 200 mM NaCl. Following treatment with ABA, *LbMAPK10*, *LbMAPK12*, and *LbMAPK18* were all upregulated compared with the control (0 h), reaching their highest levels at 48 h into treatment; by contrast, *LbMAPK16* and *LbMAPK20* were downregulated under this treatment (Fig. 6A). In response to PEG, *LbMAPK10*, *LbMAPK18*, and *LbMAPK19* were upregulated compared with the control (0 h); however, the expression of seven genes (*LbMAPK2*, *LbMAPK5*, *LbMAPK7*, *LbMAPK8*, *LbMAPK14*, *LbMAPK17*, and *LbMAPK20*) was significantly downregulated compared with their expression before treatment (Fig. 6B). In the NaCl treatment, the expression levels of *LbMAPK10*, *LbMAPK12*, and *LbMAPK18* peaked at 4 h into treatment, but *LbMAPK13*, *LbMAPK16*, and *LbMAPK20* were downregulated compared with the control (0 h) and reached their lowest levels at 24 h, 16 h, and 16 h into NaCl treatment, respectively (Fig. 6C).

### LbMAPK2 and LbMAPK10 positively regulate salt gland development in *L. bicolor*

**Fig. 7:**
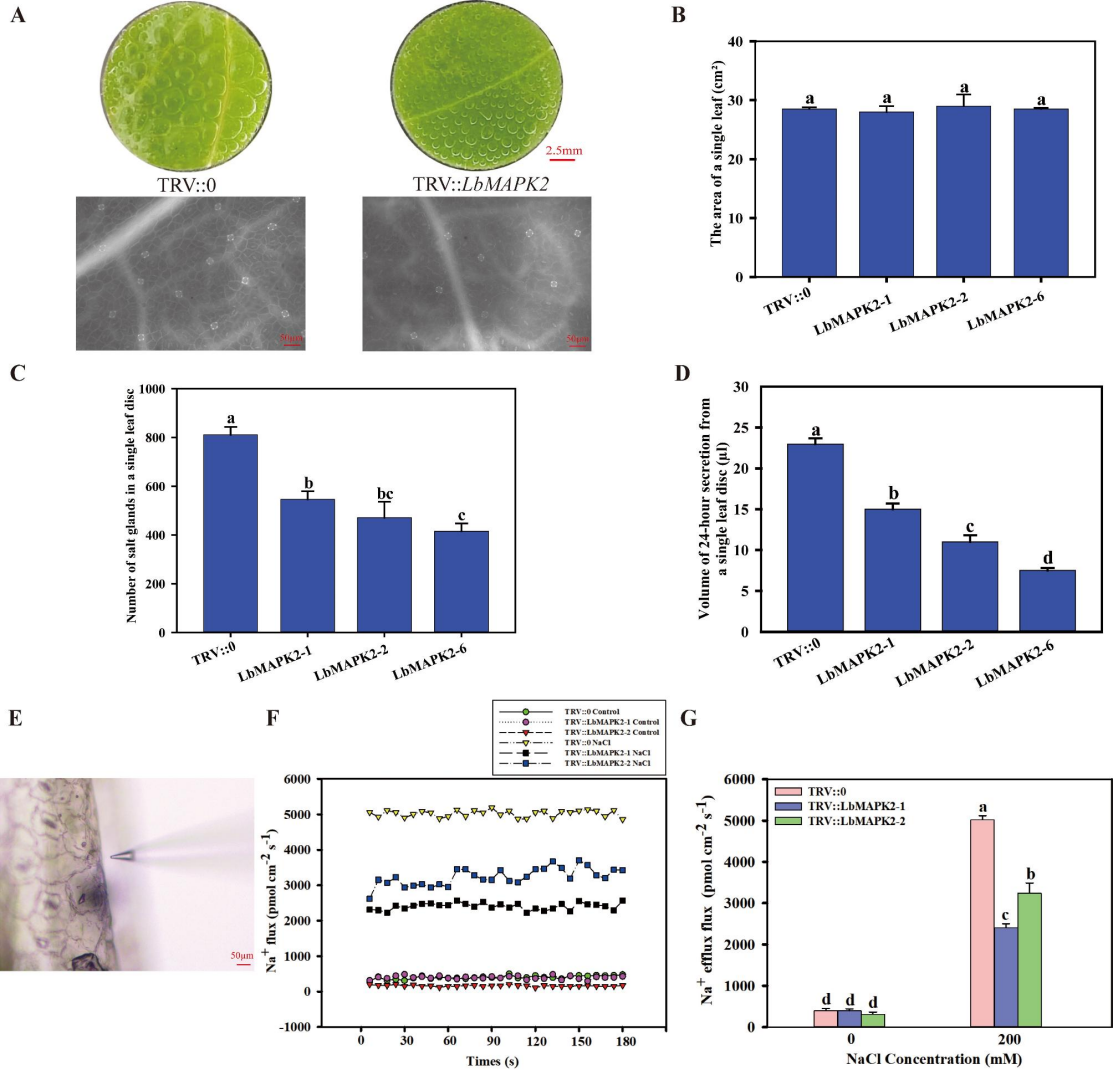
Effect of *LbMAPK2* silenceing on salt gland development and salt secretion of *L. bicolor.***(A)** Distribution and salt secretion of salt glands in different lines leaves for control group and experimental group, the scale bar of leaf disc = 2.5 mm, the scale bar of salt gland density = 50 μm. **(B)** The area of a single leaf of TRV::0 for control group and TRV::LbMAPK2 of different lines for experimental group. **(C)** The number of salt glands in TRV::0 leaf disc for control group, TRV::LbMAPK2 leaf disc for experimental group. **(D)** 24-h secretion of leaf disc of TRV::0 for control group, TRV::LbMAPK2 of different lines for experimental group. **(E)** Representative images of the non-invasive electrode used for NMT. **(F)** The Na^+^ efflux in including empty vector (TRV::0) and silenced lines of LbMAPK2. Net Na^+^ efflux rate per single salt gland of different lines over 180 s. **(G)** Average net Na^+^ efflux rate per salt gland in different lines. The data are the mean of the Na^+^ efflux based on the 180 s time course from three salt glands. All of the above data were set with three replicates and standard deviation (SD) was calculated to ensure accuracy. Letters above the bar (a-f) were used to indicate significant differences between different columns (*p* = 0.05, Duncan)

**Fig. 8:**
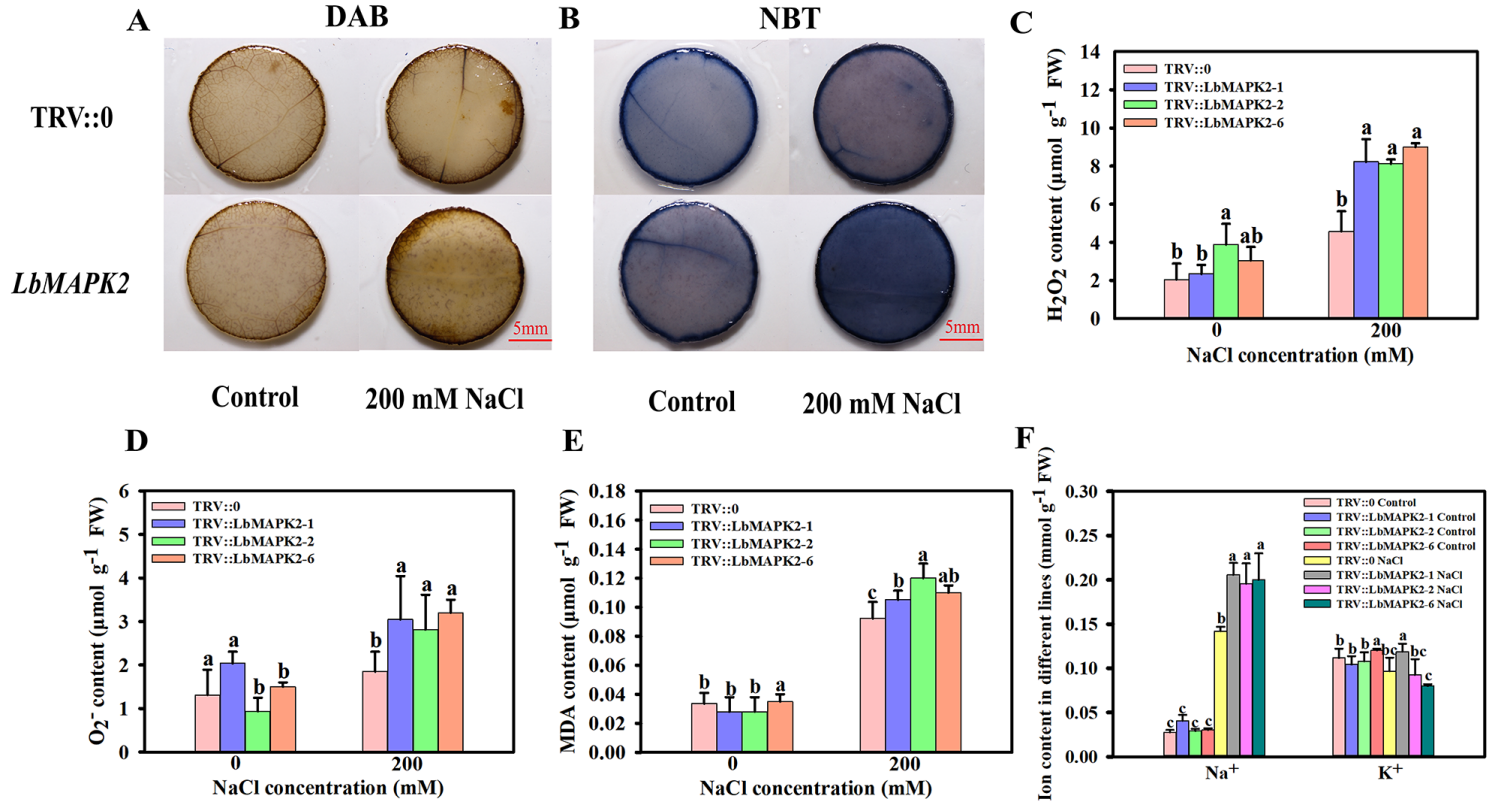
Effect of *LbMAPK2* silenceing on salt tolerance of *L. bicolor*. **(A-B)** DAB and NBT staining results of leaf discs from the TRV::0 and TRV::*LbMAPK2* in different silenced lines under normal growth conditions and under NaCl treatment. **(C-E)** Represents the content of H_2_O_2_, O_2_^·–^ and MDA in the TRV::0 and TRV::LbMAPK2 of different silenced lines respectively. **(F)** Na^+^ and K^+^ contents in the TRV:: 0 and TRV::*LbMAPK2* of different silenced lines. All of the above data were set with three replicates and standard deviation (SD) was calculated to ensure accuracy. Different letters (a-c) were used to indicate significant differences between different columns at *p* = 0.05

Based on the expression pattern, we found that *LbMAPK2* was highly expressed in the early stage of salt gland development (stage A and stage B) (Fig. 5), wihle the expression level of *LbMAPK10* was up-regulated under NaCl stress (Fig. 6C). Therefore, these two genes were chosen for further study. As virus-induced gene silencing (VIGS) has been successfully applied in *L. bicolor*, we employed VIGS to investigate the roles of LbMAPK2 (Figs. 7 and 8) and LbMAPK10 in *L. bicolor* (Figure S2, 3). We used the six-leaf stage of wild-type *L. bicolor* for VIGS. Using the empty plasmid TRV:0 as control, we individually infiltrated the constructs pTRV:*LbMAPK2* and pTRV:*LbMAPK10* into the leaves of *L. bicolor.* We collected newly emerged leaves of infiltrated plants 2 weeks after initial infiltration and performed reverse transcription–quantitative PCR (RT–qPCR) to examine the efficiency of silencing. We observed a high silencing efficiency in pTRV:*LbMAPK2* (approximately 70–80% lower expression than the control) and pTRV:*LbMAPK10* (approximately 80–85% lower expression than the control) plants (Figures S4, 5). To test whether LbMAPK2 and/or LbMAPK10 contribute to salt secretions from *L. bicolor* leaves, we harvested leaf discs (1 cm in diameter) from the wild type and different VIGS plants and placed them on top of a 200-mM NaCl solution (adaxial side of leaf discs was in contact with NaCl solution) [18, 20]. After 24 h, we carefully collected the secretions from individual leaf discs and measured the resulting volume of secretions. We obtained smaller total secretion volumes from single leaf discs harvested from silenced plants than from those harvested from wild-type plants, reaching volumes of only about 20–65% those of the wild type (Fig. 7A, D; Figure S2A, D). We also inspected and scored the density of salt glands on the abaxial side of the leaf epidermis from wild-type, *LbMAPK2-*silenced, and *LbMAPK10-*silenced plants using fluorescence microscopy. We counted 30–50% fewer salt glands per unit area in pTRV:*LbMAPK2* and pTRV:*LbMAPK10* leaves compared with wild-type leaves (Fig. 7A, C; Figure S2A, C). Importantly, total leaf area in pTRV:*LbMAPK2* and pTRV:*LbMAPK10* plants was comparable to that of leaves from control plants infiltrated with the pTRV:0 empty vector, indicating that the lower density of salt glands was not accompanied by a change in leaf area (Fig. 7B; Figure S2B).

Salt glands play an important role in salt secretion in halophytes by excreting excess Na^+^ to avoid salt damage [23]. As we counted fewer salt glands on pTRV:*LbMAPK2* and pTRV:*LbMAPK10* leaves, we wondered whether the rate of salt secretion from individual salt glands might be affected. Hence, we used non-invasive micro-test technique (NMT) to precisely measure the Na^+^ efflux from single salt glands from empty vector control (pTRV:0), pTRV:*LbMAPK2*, and pTRV:*LbMAPK10* plants grown under control conditions or exposed to 200 mM NaCl (Fig. 7E; Figure S2E). We observed no significant difference in the Na^+^ efflux rate of single salt glands from the empty vector control (pTRV:0), pTRV:*LbMAPK2*, and pTRV:*LbMAPK10* plants in the absence of NaCl. By contrast, under NaCl treatment, the Na^+^ efflux rate from single salt glands of pTRV:*LbMAPK2* and pTRV:*LbMAPK10* plants was significantly lower than that of pTRV:0 control plants (Fig. 7F, G; Figure S2F, G). We conclude that the smaller total volumes of salt secretions in *LbMAPK2-*silenced and *LbMAPK10-*silenced plants results from a combination of fewer salt glands and a lower ion efflux rate from single salt glands, indicating that LbMAPK2 and LbMAPK10 positively regulate the development of salt glands.

### LbMAPK2 and LbMAPK10 positively regulate the salt tolerance of *L. bicolor*

As the silencing of *LbMAPK2* or *LbMAPK10* via VIGS resulted in a lower salt gland density and decreased salt secretion ability from single salt glands, we speculated that the salt tolerance of pTRV:*LbMAPK2* and pTRV:*LbMAPK10* plants might be lower as well. To test this idea, we irrigated different pTRV:*LbMAPK2* and pTRV:*LbMAPK10* plants alongside pTRV:0 control plants with 200 mM NaCl for 10 d, after which we visualized plasma membrane damage caused by H_2_O_2_ and O_2_^·–^ accumulation on leaves using nitro tetrazolium blue chloride (NBT) and 3,3’-diamino benzidine (DAB) staining. We observed darker NBT and DAB staining patterns in leaf discs collected from *LbMAPK2-* and *LbMAPK10-*silenced plants compared with those in pTRV:0 leaf discs under salt treatment, but not control conditions (Fig. 8A, B; Figure S3A, B). In addition, we measured physiological indexes of salt tolerance such as endogenous reactive oxygen species contents (H_2_O_2_ and O_2_^·–^), malondialdehyde (MDA), and ion contents (Na^+^ and K^+^) in these plants. Compared with pTRV:0, the contents of H_2_O_2_, O_2_^·–^, MDA, and Na^+^ increased significantly in the pTRV:*LbMAPK2* and pTRV:*LbMAPK10* plants upon treatment with 200 mM NaCl (Fig. 8C–F; Figure S3C–F). These results demonstrate that LbMAPK2 and LbMAPK10 positively regulate the salt tolerance of *L. bicolor*.

## Discussion

The *MAPK* gene family has been described in many herb/grass plants and comprises 16 *MAPK*s in Tartary buckwheat [11], 17 in rice [24], 19 in maize [10], 20 in Arabidopsis [5], and 20 in barley (*Hordeum vulgare*) [25]. In this study, we identified 20 *MAPK*s in *L. bicolor* (Table 1), suggesting that these plant species employ similar numbers of MAPK proteins to regulate their growth and development, and to cope with various stresses. Similar to those of other species, LbMAPKs from *L. bicolor* are divided into four groups (A–D) according to their structure (Fig. 1). As in Arabidopsis, rice, and Tartary buckwheat, LbMAPK members of groups A and B all contain the TEY motif in their activation loop, whereas members of group D contain the TDY motif [5, 11, 24]. However, among the members of group C, LbMAPK12 and LbMAPK13 harbor the TDY motif, LbMAPK18 contains the TEY motif, and LbMAPK19 contains the TNY motif, which differs from other species [5, 11]. Previous studies have described other activation loop motifs in some plant MAPKs, such as TSY, TQY, TVY [26, 27], and MEY motifs [28]. We only identified a TNY (Thr-Asn-Tyr) motif in LbMAPK19; we speculate that LbMAPK19 may play a special role in the salt tolerance of *L. bicolor*, whose function remains to be studied. In addition, most LbMAPKs clustered most closely with their homologs from Tartary buckwheat, a result that is largely consistent with the results of Yuan et al. [22].

The patterns of exons and introns in genes may help us gain a better understanding of their evolutionary history [29–31]. For example, among the 16 *FtMAPK*s identified in Tartary buckwheat [11], the number of introns varied from 2 to 11, with the number of exons varying from 2 to 11 (Fig. 3). Among the 18 *LsMAPK*s in lettuce (*Lactuca sativa*), the number of introns ranged from 1 to 10 and the number of exons from 2 to 11 [32]. The number of introns among the 20 *LbMAPK*s identified in our study ranged from 1 to 10, and the number of exons from 2 to 11. Thus, the number of introns in *LbMAPK* genes is similar to that in *LsMAPK*s, but fewer than that in *FtMAPK*s. We hypothesize that the gene structure of *MAPK* family genes is conserved in different species.

*L. bicolor* is a typical recretohalophyte, which can secrete excess salt ions through its salt glands to reduce salt damage [33–35]. The salt tolerance of *L. bicolor* is related to the development of salt glands and the salt secretion ability of these salt glands [36]. The development of salt glands can be divided into five stages, with genes that are highly expressed in the early stages being postulated to regulate the development of salt glands [21]. Transcriptome data showed that *LbMAPK1*, *LbMAPK2*, *LbMAPK16*, and *LbMAPK20* are highly expressed in the early stages of salt gland development, suggesting that these genes participate in their development (Fig. 5).

Plant MAPK signaling cascades are widely involved in responses to abiotic stress, such as drought and high salinity [37–39]. For example, overexpressing *OsMAPK5* in rice increases OsMAPK5 kinase activity and salt tolerance [40]. Zhang et al. [41] showed that OsMAPK3 and ZINC FINGER PROTEIN 213 (OsZFP213) regulate rice salt tolerance by improving the ability to scavenge reactive oxygen species. In Arabidopsis, heterologously expressing the *ZmMAPK1* gene from maize can increase proline content in transgenic plants experiencing drought stress, decrease their MDA content, and prevent the production of reactive oxygen species under high-temperature stress [42]. Expressing maize *Salt-induced mitogen-activated protein kinase 1* (*ZmSIMK1*) in Arabidopsis increases the tolerance of transgenic lines to salt stress [43]. As an extreme halophyte, *L. bicolor* responds to salt stress by upregulating the expression of many genes to adjust to the saline environment [21]. ABA is a stress signal that plays an important role in plant stress responses and tolerance [44]. The proteins encoded by MAPKs can respond to ABA, drought stress, and salt stress [45]. Our results showed that *LbMAPK10*, *LbMAPK12*, and *LbMAPK18* were upregulated upon ABA treatment; *LbMAPK10*, *LbMAPK18*, and *LbMAPK19* were upregulated under PEG treatment; and *LbMAPK10*, *LbMAPK12*, *LbMAPK13*, *LbMAPK16*, *LbMAPK18*, and *LbMAPK20* were upregulated by NaCl treatment (Fig. 6). These results indicate that these *MAPK* genes play an important role in the response to abiotic stress in *L. bicolor*.

**Fig. 9:**
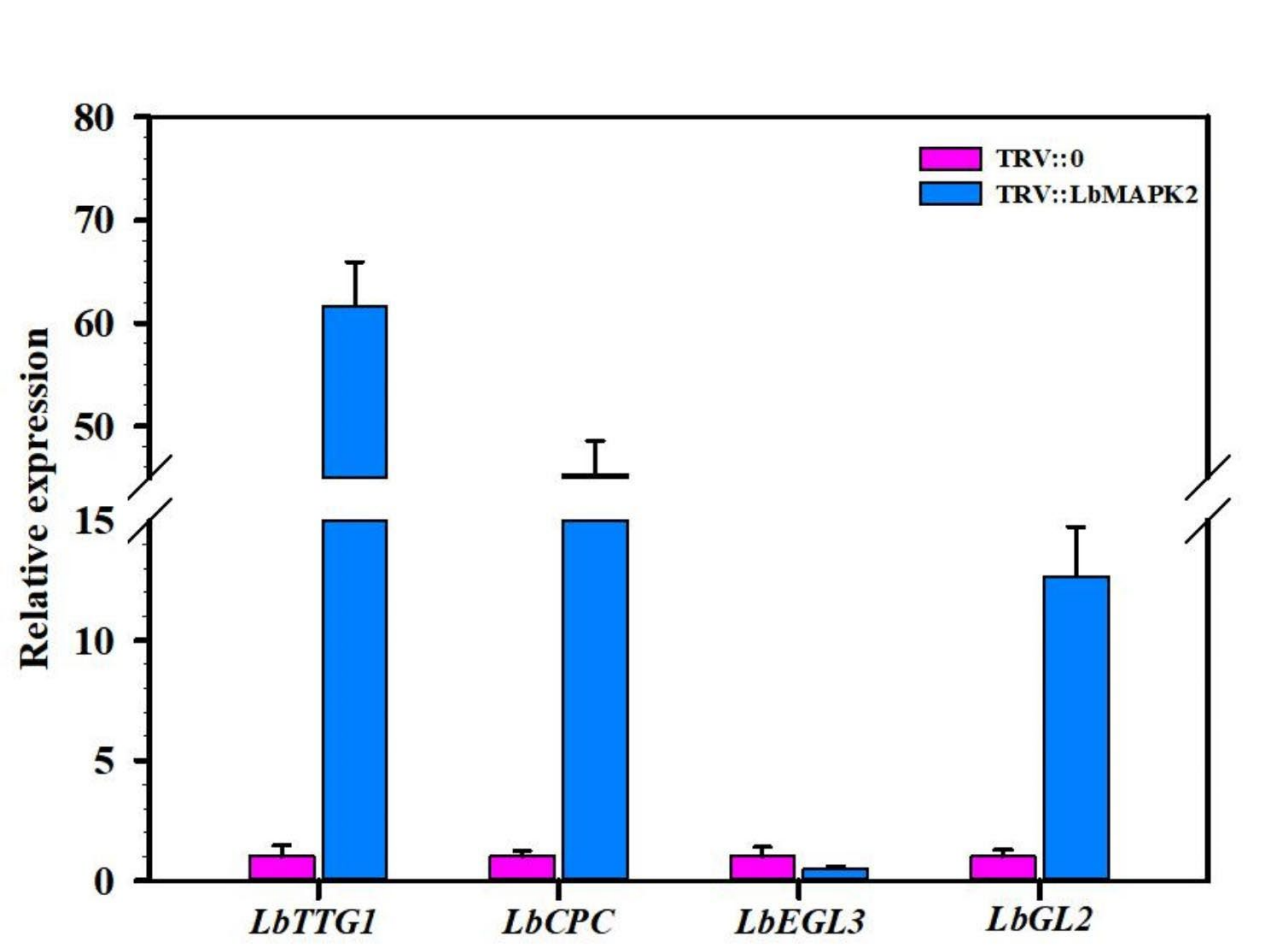
Expression of genes associated with salt gland development in *LbMAPK2* silenced line. Different color blocks represent different lines

Many genes related to salt gland development have been identified in *L. bicolor*. Yuan et al. [21] isolated mutants with a high density of salt glands following gamma ray irradiation, providing excellent materials for studying the expression levels of genes predicted to participate in salt gland development. Indeed, the expression levels of *TRANSPARENT TESTA OF GL1* (*LbTTG1*), *CAPRICE* (*LbCPC*), and *GLABRA2* (*LbGL2*) in these mutants are significantly downregulated relative to wild-type levels, indicating that these genes negatively regulate salt gland development. By contrast, *ENHANCER OF GL3* (*LbEGL3*) is significantly upregulated, suggesting that LbEGL3 positively regulates salt gland development. Yuan et al. [22] showed that LbTTG1 and LbHLH, a basic helix-loop-helix transcription factor, physically interact to negatively regulate salt gland development; moreover, *LbTTG1*-knockout lines have a higher salt gland density than the wild type. Zou et al. [46] showed that LbCPC negatively regulates salt gland development in *L. bicolor*. Salt gland density in lines overexpressing *LbCPC* is significantly lower than that of the wild type, whereas plants with *LbCPC* silenced through VIGS show higher salt gland density than the wild type. In our study, the salt gland density of *LbMAPK2*-silenced plants was significantly lower than that of the empty vector control, as was the salt secretion ability of leaves (Fig. 7). Further analysis showed that the expression of negative regulators of salt gland development (*LbTTG1*, *LbCPC*, and *LbGL2*) was substantially upregulated in *LbMAPK2*-silenced plants, while the expression of a positive regulator of salt gland development, *LbEGL3*, was significantly downregulated (Fig. 9). Therefore, LbMAPK2 may regulate salt gland development by regulating the expression of the above genes. *LbMAPK2*-silenced plants show a lower salt secretion ability, preventing much Na^+^ from being excreted from plant cells and causing it to accumulate in leaves; this damages the cells, as evidenced by the large amount of reactive oxygen species produced, resulting in oxidative stress [47]. Han et al. [20] showed that VIGS of *LbMYB48* results in a lower salt gland density than that in empty vector control plants, leading to a diminished salt tolerance and higher accumulation of Na^+^, H_2_O_2_, O_2_^·–^, and MDA. In our study, *LbMAPK2*-silenced plants also had a low salt gland density and salt excretion ability, resulting in a corresponding increase in Na^+^, H_2_O_2_, O_2_^·–^, and MDA contents in their leaves under salt stress and decreased salt tolerance (Fig. 8).

In summary, we identified 20 MAPK family members in *L. bicolor* and demonstrated that several *LbMAPK*s respond to ABA treatment and/or to PEG or NaCl treatment. *LbMAPK2* is highly expressed in the early stages of salt gland development and promotes salt gland development and tolerance to high salinity by regulating the expression of salt gland development-related genes. As MAPKs can phosphorylate downstream transcription factors or other small proteins, and can themselves be phosphorylated by upstream MAPKKs [2, 48], a phosphoproteomics analysis would be interesting to further study the stress tolerance function of MAPK family members in *L. bicolor*.

## Materials and methods

### Identification of candidate *MAPK*s in *L. bicolor*

Genomic data for *Limonium bicolor* was obtained from NCBI (https://www.ncbi.nlm.nih.gov/), and keyword searches were employed to search for MAPKs in the sequenced and annotated genome. Analysis of conserved domains was performed at NCBI with the CD search tool (https://www.ncbi.nlm.nih.gov/Structure/bwrpsb/bwrpsb.cgi). Sequence alignment was performed at the website http://multalin.toulouse.inra.fr/multalin/. The molecular weight (MW) and isoelectric point (pI) of LbMAPKs were predicted using the ExPASY online tool (https://web.expasy.org/protparam/). Subcellular localization was predicted online using the tool http://www.csbio.sjtu.edu.cn/bioinf/Cell-PLoc-2/.

### Phylogenetic analysis and chromosomal location of MAPK family members in *L. bicolor*

The amino acid sequences of LbMAPKs were aligned using MEGA 11.0 software (Table S1). A phylogenetic tree was reconstructed using MAPK sequences from four plant species [Arabidopsis (*Arabidopsis thaliana*), rice (*Oryza sativa*), Tartary buckwheat (*Fagopyrum tataricum*), and *Limonium bicolor*] following the maximum neighbor-joining (NJ) method with 1000 bootstraps. TBtools was used to visualize the distribution of *LbMAPK* genes on chromosomes [49].

### Structural analysis of MAPKs in *L. bicolor*

The MEME tool was used to identify up to 15 conserved motifs in the full-length protein sequences of LbMAPKs (https://meme-suite.org/tools/meme) [50]. Conserved domains were also identified through the conserved domain database search tool of NCBI (https://www.ncbi.nlm.nih.gov/cdd/?term=). To analyze the *cis*-regulatory elements in the promoters of *LbMAPK* genes, the 2.2-kb sequence upstream of each *LbMAPK* gene was analyzed using the PlantCARE database (http://bioinformatics.psb.ugent.be/webtools/plantcare/html/). TBtools was used to visualize the protein motifs and *cis*-acting elements.

### Plant materials and abiotic stress treatment

*L. bicolor* seedlings were grown in a growth chamber, under conditions described by Li et al. [51], at the College of Life Sciences, Shandong Normal University in Jinan, Shandong, China. Abiotic stress treatments [200 mM NaCl, 20% (w/v) polyethylene glycol 6000 (PEG) (Kermel, China), or 100 µM ABA (Biotopped, China)] were performed according to the method of Zhang et al. [52]. Fresh leaves were collected immediately before (0 h) and at 4 h, 8 h, 12 h, 24 h, and 48 h after the onset of treatment, frozen in liquid nitrogen, and stored at − 80℃ until use.

### Reverse transcription–quantitative PCR (RT–qPCR)

Total RNA was extracted from the above samples using a FastPure Plant Total RNA Isolation Kit (Vazyme, China). First-strand cDNAs were synthesized using an Evo M-MLV RT Mix Kit with gDNA Clean for qPCR (Accurate Biology, China). The qPCR primers (Table S2) were designed using Beacon Designer 7 software. Reference genes (*Tubulin*) were selected according to Zhang et al. [36]. A SYBR Green Premix *Pro Taq* HS qPCR Kit (Accurate Biology, China) was used for qPCR. The amplification program used was as follows: 95 °C for 30 s followed by 40 cycles of 95 °C for 5 s and 60 °C for 30 s. Gene expression levels were calculated using the 2^–∆∆CT^ method [53]. Primers for genes related to salt gland development were in Table S3.

### Transcriptome data

Transcriptome data can be obtained from the https://www.ncbi.nlm.nih.gov/sra/?term=Limonium%20Bicolor%20 website.

### Acquisition of gene silencing lines via virus-induced gene silencing (VIGS)

The pTRV2-*LbMAPK2* plasmid was constructed by inserting specific fragments from *LbMAPK2* into the pTRV2 plasmid. pTRV2, pTRV2-*LbMAPK2*, and the assistant infection plasmid pTRV1 were individually transformed into Agrobacterium (*Agrobacterium tumefaciens*) strain GV3101; a mixed bacterial suspension harboring pTRV1 and pTRV2-*LbMAPK2* was used as the experimental group. Bacterial suspensions carrying pTRV1 and pTRV2 were mixed in the control group. Each bacterial suspension was infiltrated into the leaves of 45-day-old *L. bicolor* plants according to Han et al. [20]. After 2 days of dark incubation, infiltrated plants were exposed to light. Newly grown leaves were sampled approximately 2 weeks later [20, 54]. Silencing of *LbMAPK10* followed the same method.

### Measurements of malondialdehyde (MDA), H_2_O_2_, and O_2_^·–^ contents

MDA content in leaves of different *L. bicolor* lines was determined by spectrophotometric determination [55] as detailed below. Leaves were collected from the same leaf position of different genotypes. The leaves (0.1 g) were ground thoroughly, after which 0.75 mL 0.1% (w/v) tricarboxylic acid was added, followed by adding 1.25 mL 0.5% (w/v) thiobarbituric acid (TCA) solution. The total volume of the reaction solution was 2 mL. The samples were mixed well and boiled for 10 min. The tubes were allowed to cool down on ice before being centrifuged at 3000 g for 15 min at 25℃. The absorbance of the supernatant was measured at 532 and 600 nm. The formula for calculating MDA content in leaves was based on that reported by Han et al. [20].

The contents of H_2_O_2_ and O_2_^·–^ in the leaves of different *L. bicolor* plants was determined using a UV spectrophotometer (UV756, Shanghai Youke). To determine H_2_O_2_ content, leaves (0.1 g fresh weight) were ground, mixed with 5 mL acetone at 4℃, and transferred to a centrifuge tube. Samples were centrifuged at 3000 g for 8 min at 25℃, yielding the supernatant as the test solution. To each 1 mL test solution, 0.1 mL 20% (w/v) TiCl_4_ and 0.2 mL concentrated ammonia was added, followed by centrifugation at 5000 g for 7 min at 25℃. The precipitate was washed with acetone 3–5 times and then dissolved with 5 mL 2 M H_2_SO_4_ and diluted to 10 mL. The absorbance of the resulting solution was measured at 415 nm, and the H_2_O_2_ content was calculated according to Li et al. [47]. To determine O_2_^·–^ content, leaves (0.5 g fresh weight) were quickly ground on ice with 50 mM phosphate buffered saline (PBS, pH 7.8), and the supernatant was retained for later use. The absorbance was measured at 530 nm. The O_2_^·–^ content was calculated according to Li et al. [47].

### Measurement of Na^+^ and K^+^ contents

Plants from different lines of *L. bicolor* were treated with 200 mM NaCl or 0 mM NaCl (control), and the corresponding leaves at the same leaf position were harvested 10 days after the onset of treatment. The contents of Na^+^ and K^+^ were determined using a flame photometer (JC-YZ-600, China) [56].

### Measurement of Na^+^ efflux rate in a single salt gland

Salt glands on the epidermis were identified by using the NMT method under a microscope, and the Na^+^ efflux rate of the salt glands was measured using NMT (NMT100-SIM-XY, Younger USA, MA 01002, USA) as previously described [22, 57]. Briefly, the test solution contained 0.5 mM NaCl and 0.1 mM CaCl_2_ (pH 6.0). The calibration liquid contained 0.1 mM NaCl and 1 mM NaCl (pH 6.0). The filling liquid was 250 mM NaCl. The Na^+^ efflux rate of a single salt gland was recorded every 10 s over 180 s. The average Na^+^ efflux was calculated from data of three salt glands over 180 s.

### Statistical analysis

All experimental data were visualized in Sigmaplot 12.5, and the statistical significance was determined at the 0.05 and 0.01 probability levels by analysis of variance (ANOVA) in IBM SPSS Statistics 26.0. Each of the above experimental results was calculated from three replicates.

### Electronic supplementary material

Below is the link to the electronic supplementary material.

Additional file 1: Table S1. List of the 20 MAPK genes identified in this study

Additional file 2: Table S2. The primers for 20 MAPK genes used in real-time qPCR analysis.

Additional file 3: Table S3. Primers of genes associated with salt gland development.

Additional file 4: Figure S1. Conserved structure motif.

Additional file 5: Figure S2. Effect of LbMAPK10 silenceing on salt gland development and salt secretion of L. bicolor.

Additional file 6: Figure S3. Effect of LbMAPK10 silenceing on salt tolerance of L. bicolor.

Additional file 7: Figure S4. The expression level of different lines of LbMAPK2.

Additional file 8: Figure S5. The expression level of different lines of LbMAPK10.

Supplementary Material 9

## Data Availability

Not applicable.

## Abbreviations

MAPK: Mitogen-activated protein kinase
H_2_O_2_: Hydrogen peroxide
MDA: Malondialdehyde
O_2_^·–^: Superoxide anion
ROS: Active oxygen species
