# Supplementary figures and images for: Genome-wide identification of the mitogen-activated kinase gene family from Limonium bicolor and functional characterization of LbMAPK2 under salt stress
Source: BMC Plant Biol. 2023 Nov 15;23:565. doi: 10.1186/s12870-023-04589-x (PMC10647163; doi:10.1186/s12870-023-04589-x)

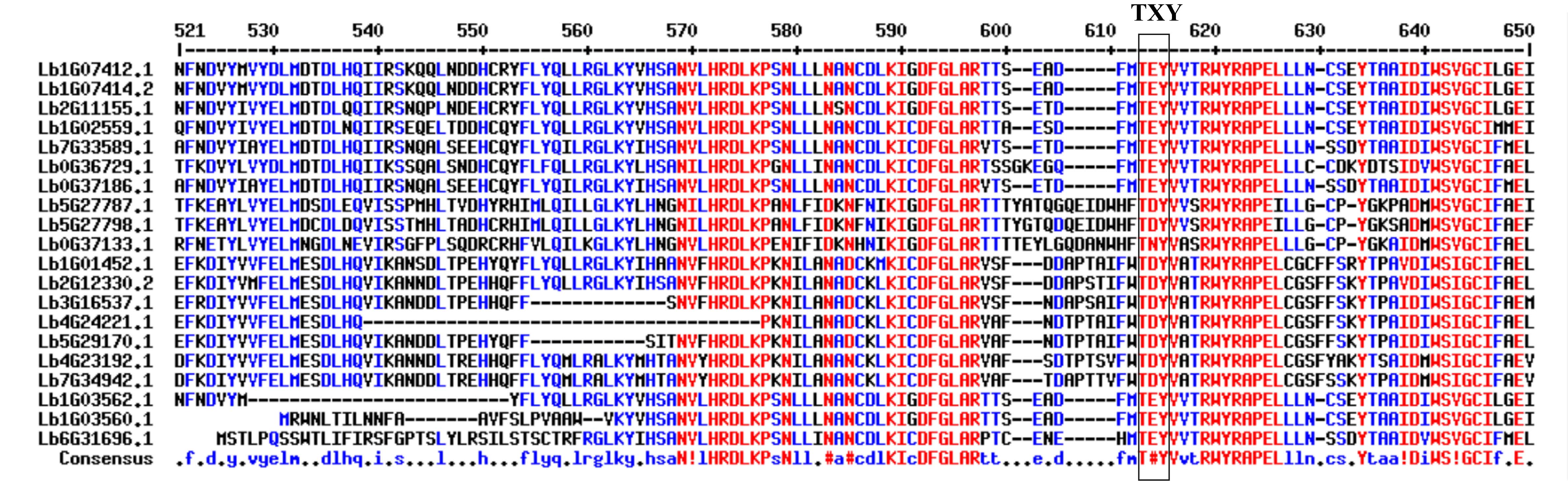

Supplement: Supplementary file 1 — Additional file 1: Table S1. List of the 20 MAPK genes identified in this study [file 12870_2023_4589_MOESM1_ESM.jpg]

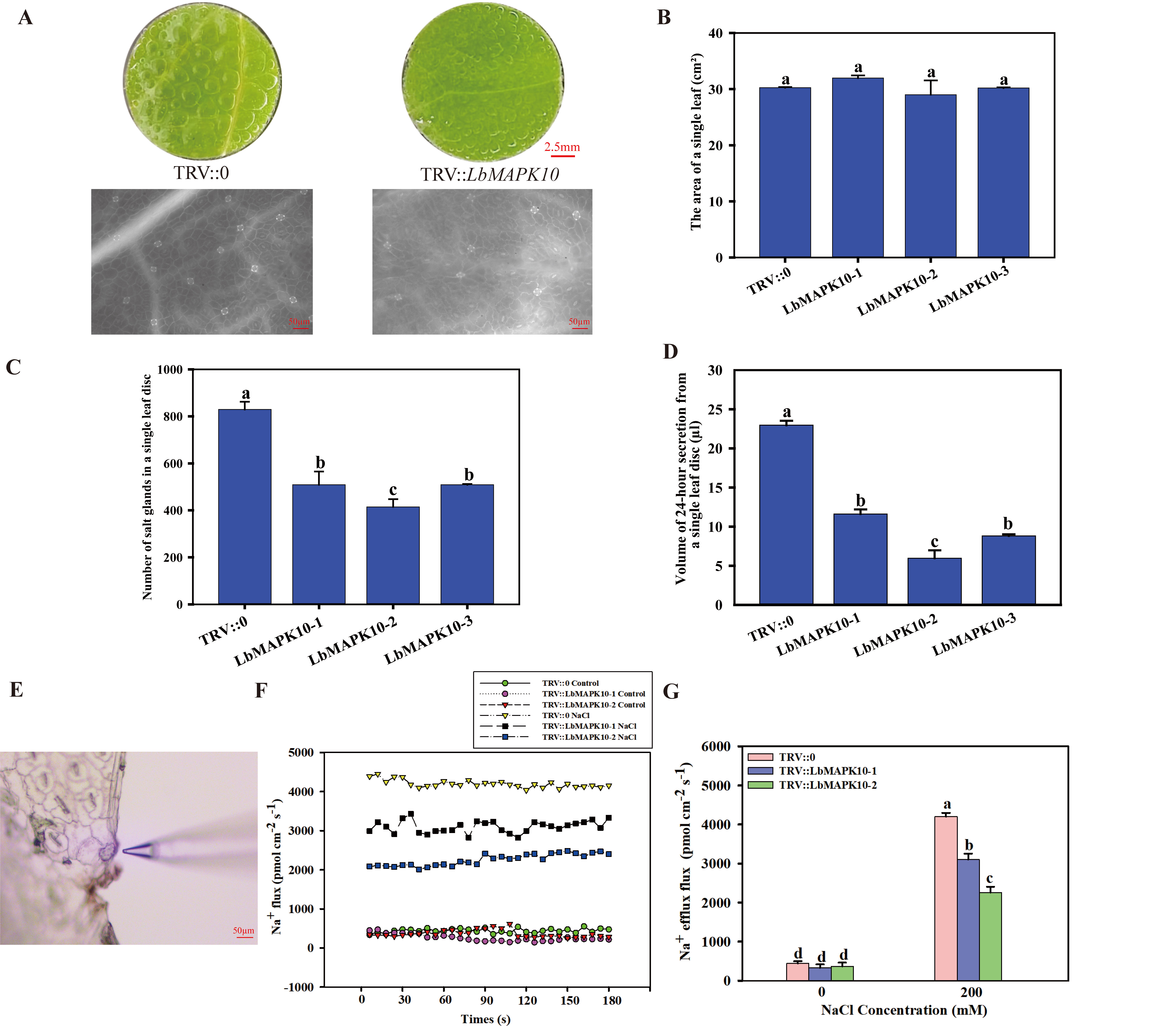

Supplement: Supplementary file 2 — Additional file 2: Table S2. The primers for 20 MAPK genes used in real-time qPCR analysis. [file 12870_2023_4589_MOESM2_ESM.jpg]
