# Supplementary material for: Genome-wide identification of the mitogen-activated kinase gene family from Limonium bicolor and functional characterization of LbMAPK2 under salt stress
Source: BMC Plant Biol. 2023 Nov 15;23:565. doi: 10.1186/s12870-023-04589-x (PMC10647163; doi:10.1186/s12870-023-04589-x)
Supplement: Supplementary file 3 — Additional file 3: Table S3. Primers of genes associated with salt gland development. [file 12870_2023_4589_MOESM3_ESM.docx]

**Supplementary Figure Legends**

**Figure S1:** The conserved structure motif of 20 LbMAPK proteins from *L. bicolor*. The conserved amino acid signature motif T×Y is highlighted in black box.

**Figure S2：**Effect of *LbMAPK10* silenceing on salt gland development and salt secretion of *L. bicolor.* (A) Distribution and salt secretion of salt glands in different lines leaves for control group and experimental group, the scale bar of leaf disc = 2.5 mm, the scale bar of salt gland density = 50 µm. (B) The area of a single leaf of TRV::0 for control group and TRV::LbMAPK10 of different lines for experimental group. (C) The number of salt glands in TRV::0 leaf disc for control group, TRV::LbMAPK10 leaf disc for experimental group. (D) 24-h secretion of leaf disc of TRV::0 for control group, TRV::LbMAPK10 of different lines for experimental group. (E) Representative images of the non-invasive electrode used for NMT. (F) The Na^+^ efflux in including empty vector (TRV::0) and silenced lines of LbMAPK10. Net Na^+^ efflux rate per single salt gland of different lines over 180 s. (G) Average net Na^+^ efflux rate per salt gland in different lines. The data are the mean of the Na^+^ efflux based on the 180 s time course from three salt glands. All of the above data were set with three replicates and standard deviation (SD) was calculated to ensure accuracy. Letters above the bar (a-f) were used to indicate significant differences between different columns (*p* = 0.05, Duncan).

**Figure S3：**Effect of *LbMAPK10* silenceing on salt tolerance of *L. bicolor*. (A-B) DAB and NBT staining results of leaf discs from the TRV::0 and TRV::*LbMAPK10* in different silenced lines under normal growth conditions and under NaCl treatment. (C-E) Represents the content of H_2_O_2_, O_2_^·–^ and MDA in the TRV::0 and TRV::LbMAPK10 of different silenced lines respectively. (F) Na^+^ and K^+^ contents in the TRV:: 0 and TRV::*LbMAPK10* of different silenced lines. All of the above data were set with three replicates and standard deviation (SD) was calculated to ensure accuracy. Different letters (a-c) were used to indicate significant differences between different columns at *p* = 0.05.

**Figure S4:** The expression level of *LbMAPK2* in different silencing lines. Letters above the bar (a-e) were used to indicate significant differences between different columns (*p* = 0.05, Duncan).

**Figure S5:** The expression level of *LbMAPK10* in different silencing lines. Letters above the bar (a-e) were used to indicate significant differences between different columns (*p* = 0.05, Duncan).
