# Supplementary material for: Genome-wide identification of the mitogen-activated kinase gene family from Limonium bicolor and functional characterization of LbMAPK2 under salt stress
Source: BMC Plant Biol. 2023 Nov 15;23:565. doi: 10.1186/s12870-023-04589-x (PMC10647163; doi:10.1186/s12870-023-04589-x)
Supplement: Supplementary file 6 — Additional file 6: Figure S3. Effect of LbMAPK10 silenceing on salt tolerance of L. bicolor. [file 12870_2023_4589_MOESM6_ESM.docx]

**Table S1 List of the 20 *MAPK* genes identified in this study**

| **Gene name** | **Gene ID** | **Sequence Type** | **Sequences** |
| --- | --- | --- | --- |
| LbMAPK1 | Lb1G01452.1 | CDS | ATGGTTGGAGGAGGAGGAGGAGCTTTAGTCGGATTACGGCGCTGGCTCAACCGGCGAACATGCGTGAACAGCGCCGCCACCCCCGATGGTTTCAATCCCGATGATTTGCCTGAGGACTCTGCCACTTCCGTCGAGCCGCCGCCTCCACCTGCTCCGTCCTCATCGGCTTCTGCCGCGGAAGCGCTTGACTTGTCCAGTTTGAAACTGATTAAAGTTCCCAAACGCTTCAATTTCAACTTCGCCATTTCAGTAGATCCTCACAAAAAGGACATGGTTGAGAAAGAATTCTTCACTGAATATGGAGAAGCAAACCGGTATGAGATTCAAGAAGTAATTGGGAAGGGAAGCTATGGGGTTGTTGCATCTGCAATTGACACTCGTACGGGTGAAAAGGTGGCTATAAAGAAAATAAATGATGTTTTTGAGCATGTTTCTGATGCCACTCGCATTCTCAGAGAAATCAAGCTCCTTCGGCTTCTTCACCATCCTGATGTTGTTGAAATAAAGCATATCATGCTCCCTCCATCTCAAAGGGAATTCAAAGATATTTATGTGGTGTTTGAGTTAATGGAATCAGACCTACATCAAGTTATAAAAGCAAACAGTGATCTAACTCCAGAACACTACCAGTATTTCTTATATCAGCTCCTTCGAGGACTAAAATATATTCATGCAGCAAATGTGTTCCACCGGGACTTAAAGCCAAAAAATATTCTTGCTAATGCAGACTGCAAAATGAAAATATGTGATTTTGGACTTGCTCGAGTATCATTTGATGATGCGCCAACGGCTATCTTTTGGACGGATTACGTTGCCACTAGATGGTATCGAGCACCTGAGCTTTGCGGCTGTTTCTTCTCCAGGTACACACCAGCGGTTGACATCTGGAGTATCGGATGTATATTTGCTGAGTTGCTCACTGGAAAGCCTCTATTTCCTGGGAAGAATGTTGCTCATCAGTTAGATTTAATGACGAATTTGCTTGGAACCCCATCCGTGGAGTGTATTTCCCGGATTCGAAATGATAAGGCTAGAAGGTACCTAAATAATATGAGCAAAAAAGATCCGGTTCCTTTCTCCCAGAAATTTCCGAAGGCAGATCCTTTGGCTCTCCGTTTACTTGAACGATTGCTTGCGTTTGATCCTAAAGACCGCCCTTCTGCTGAAGAGGCATTGTCAGATCCATATTTTTTTGGCTTGGCTGATGTAGAAAATGAACCATCCACACAACCTATTTCAAAGATTGAGTTTGCATTTGAGTGGAAAAGGATGAAAAAGGATGATGTGAGAGAGCTTATATACAGGGAGATCTTAGAGTATCACCCACAAATGTTACACGAGCACCTCCACAGCACTAATCAACCTAGCTTTTTATATCCAAGTGGGGTTGATCGATTCAAACGCCAGTTTGCCCATCTTGAGGATCATCTTGGTAAAGGAGAAAATGCTACTCCGTCACTGCAAAGGAAACATGTATCTTTGCCTAGGGAGCGTGTGCAAGCTTCTAAGAGCGACAACGTCAATCATCATCAGGCTGGTCAATCAAAGGAGCGCATGGCTACTGTTGACAGCACGCCTCAAAAAGAGAGTGTGGATGCATCCAAAACTGATGGTAAAGACCTGAAATCACACGGTGGAAATGGGCATAGTACAAATTATGACTTCTGA |
|  |  | Protein | MVGGGGGALVGLRRWLNRRTCVNSAATPDGFNPDDLPEDSATSVEPPPPPAPSSSASAAEALDLSSLKLIKVPKRFNFNFAISVDPHKKDMVEKEFFTEYGEANRYEIQEVIGKGSYGVVASAIDTRTGEKVAIKKINDVFEHVSDATRILREIKLLRLLHHPDVVEIKHIMLPPSQREFKDIYVVFELMESDLHQVIKANSDLTPEHYQYFLYQLLRGLKYIHAANVFHRDLKPKNILANADCKMKICDFGLARVSFDDAPTAIFWTDYVATRWYRAPELCGCFFSRYTPAVDIWSIGCIFAELLTGKPLFPGKNVAHQLDLMTNLLGTPSVECISRIRNDKARRYLNNMSKKDPVPFSQKFPKADPLALRLLERLLAFDPKDRPSAEEALSDPYFFGLADVENEPSTQPISKIEFAFEWKRMKKDDVRELIYREILEYHPQMLHEHLHSTNQPSFLYPSGVDRFKRQFAHLEDHLGKGENATPSLQRKHVSLPRERVQASKSDNVNHHQAGQSKERMATVDSTPQKESVDASKTDGKDLKSHGGNGHSTNYDF* |
| LbMAPK2 | Lb1G02559.1 | CDS | ATGGAGAATGAAGCTAAAAAGGTTGAAGTGAAGGGTATACCCACACATGGAGGCAGGTATCTTCTCTACAACATTCTCGGGAATGTTTTCCAAATCTCCTCCAAATATGTTCCTCCTATGCTACCCGTTGGTCGTGGCGCTTACGGCATAGTCTGCTGTGCAAGAAACTCTGAGAATGAAGAACAGGTTGCTATTAAGAAGATTGCAGATGCTTTTAACAACAGCATTGATGCCAAAAGGACTCTTCGTGAGATCAAGCTTCTCTGCCATATGGACCATGACAACGTTGTGAAAATCAGAGACATTATACGTCCGCCTGACAAAGATCAGTTTAATGACGTTTACATCGTCTATGAATTAATGGATACTGATCTCAATCAAATAATACGCTCTGAACAGGAACTTACAGATGATCACTGCCAGTATTTTCTCTACCAACTATTACGGGGTCTGAAGTATGTACACTCTGCAAATGTTTTGCATCGAGATCTCAAGCCAAGTAATTTGCTCTTAAATGCCAACTGTGACCTCAAGATATGCGACTTTGGGTTGGCACGAACTACTGCAGAATCTGATTTTATGACTGAATATGTGGTAACCAGATGGTACCGAGCCCCTGAGCTGCTACTTAATTGTTCAGAGTATACGGCAGCAATAGATATCTGGTCAGTCGGTTGCATTATGATGGAAATTCTGAGGCGAGAGCCACTTTTTCCAGGCAAAGACTACTTGCAGCAATTGGGACTCATCAATACGCTTCTGGGATCTCCTGAAGATTCTGATCTTGGGTTCTTGAGAAGTGAAAATGCGAAGAACTATGTGAAGCAACTTCCACAGGTGCCAAAGGAGCCTTTTACACAGAAGTTTCCTGACCTGTCTCCTGTGGCACTTGATCTTGCAGAGAAAATGCTGGTCTTTGATCCCAGCAAACGCATCACCGTTGAAGAAGCTATGAACCACCCGTATATATCCAGTCTGCACGAGATCAATGAGGAGCCGACTTGCCCGGCTCCATTTGTGTTTGATTTTGAACAAGCATCTTTGACCGAGGAAGATATAAAGGAGCTCATATGGCAGGAGTCAGTGAAATTCAATCCGGATACAGAGTAA |
|  |  | Protein | MENEAKKVEVKGIPTHGGRYLLYNILGNVFQISSKYVPPMLPVGRGAYGIVCCARNSENEEQVAIKKIADAFNNSIDAKRTLREIKLLCHMDHDNVVKIRDIIRPPDKDQFNDVYIVYELMDTDLNQIIRSEQELTDDHCQYFLYQLLRGLKYVHSANVLHRDLKPSNLLLNANCDLKICDFGLARTTAESDFMTEYVVTRWYRAPELLLNCSEYTAAIDIWSVGCIMMEILRREPLFPGKDYLQQLGLINTLLGSPEDSDLGFLRSENAKNYVKQLPQVPKEPFTQKFPDLSPVALDLAEKMLVFDPSKRITVEEAMNHPYISSLHEINEEPTCPAPFVFDFEQASLTEEDIKELIWQESVKFNPDTE* |
| LbMAPK3 | Lb1G03560.1 | CDS | ATGAGATGGAATCTGACTATTTTGAATAATTTTGCTGCAGTATTTTCTCTACCAGTTGCTGCGTGGGTGAAGTATGTTCACTCTGCTAATGTGCTGCATCGTGACCTGAAACCAAGCAATCTCCTGCTGAATGCTAATTGTGACCTTAAAATTGGAGATTTTGGTCTTGCACGAACAACTTCTGAAGCAGATTTCATGACCGAGTATGTTGTGACTCGGTGGTATCGAGCTCCAGAACTGCTCCTAAATTGTTCAGAATATACAGCTGCAATTGATATCTGGTCCGTGGGTTGCATACTTGGAGAAATTATGACCAGAGAGCCTCTGTTTCCTGGCAAGGATTATGTTCACCAGCTTCGACTTATTACAGAGCTCATAGGTTCTCCTGATGACTCCAGCCTTGGATTTCTACGAAGCGATAATGCACGCAGGTATGTGAAGCAGCTTCCACAGTACCCAAAGCAGCATTTTCCTGCTAGATTCCCAAACGGATCTGCGATGGCTGTTGATTTGCTGGAAAAGATGCTTGTATTTGATCCTAACAAGCGAATAACAGTAAATGATGCTCTCTGCCACCCATATTTGGCCCCACTTCACGATATAAATGATGAGCCAGTCTGTTCCCAGCCTTTCAGTTTTGACTTTGAGAATCCTTCGATCACAGAGGAGCATATCAAGGAGCTAATTTGGAGGGAGTCGGTTAGATTCAACCCAGATCCTTGA |
|  |  | Protein | MRWNLTILNNFAAVFSLPVAAWVKYVHSANVLHRDLKPSNLLLNANCDLKIGDFGLARTTSEADFMTEYVVTRWYRAPELLLNCSEYTAAIDIWSVGCILGEIMTREPLFPGKDYVHQLRLITELIGSPDDSSLGFLRSDNARRYVKQLPQYPKQHFPARFPNGSAMAVDLLEKMLVFDPNKRITVNDALCHPYLAPLHDINDEPVCSQPFSFDFENPSITEEHIKELIWRESVRFNPDP* |
| LbMAPK4 | Lb1G03562.1 | CDS | ATGACACCAGAGACGAAATCTGGCCATGGTGCTGCCGGGCAGAACCGACACGGTAGCAACGTGAGAGGAGTACTAACTCATGGTGATCAATATGTTCAGTACAATCTGTTCGGTAACCTCTTTGAGGTCTCCAGAAAGTACGTTCCTATCCGCCCTGTCGGTCGTGGAGCTTACGGTATTGTTTGTGCTGCTGTGAATTCAGAAACACGGGAAGAAGTTGCGATCAAGAAGGTTGGCAATGCTTTTGATAATAGAATAGATGCTAAGAGGACATTACGTGAAATAAAGCTTCTACGCTACTTGCGCCATGAAAACGTGATTGCCTTATTGGACGTTATTCGACCTCCTCAGAGGGAGAACTTTAATGATGTATACATGTATTTTCTCTACCAGTTGCTGCGTGGTTTGAAGTATGTTCACTCTGCTAATGTGCTGCATCGTGACCTGAAACCAAGCAATCTCCTGCTGAATGCTAATTGTGACCTTAAAATTGGAGATTTTGGTCTTGCACGAACAACTTCTGAAGCAGATTTCATGACCGAGTATGTTGTGACTCGGTGGTATCGAGCTCCAGAACTGCTCCTAAATTGTTCAGAATATACAGCTGCAATTGATATCTGGTCCGTGGGTTGCATACTTGGAGAAATTATGACCAGAGAGCCTCTGTTTCCTGGCAAGGATTATGTTCACCAGCTTCGACTTATTACAGAGCTCATAGGTTCTCCTGATGACTCCAGCCTTGGATTTCTACGAAGCGATAATGCACGCAGTTTTGACTTTGAGAATCCTTCGATCACAGAGGAGCATATCAAGGAGCTAATTTGGAGGGAGTCGGTTAGATTCAACCCAGATCCTTGA |
|  |  | Protein | MTPETKSGHGAAGQNRHGSNVRGVLTHGDQYVQYNLFGNLFEVSRKYVPIRPVGRGAYGIVCAAVNSETREEVAIKKVGNAFDNRIDAKRTLREIKLLRYLRHENVIALLDVIRPPQRENFNDVYMYFLYQLLRGLKYVHSANVLHRDLKPSNLLLNANCDLKIGDFGLARTTSEADFMTEYVVTRWYRAPELLLNCSEYTAAIDIWSVGCILGEIMTREPLFPGKDYVHQLRLITELIGSPDDSSLGFLRSDNARSFDFENPSITEEHIKELIWRESVRFNPDP* |
| LbMAPK5 | Lb1G07412.1 | CDS | ATGACACCAGAGACGAAATCTGGCCATGGTGCTGCCGGGCAGAACCGACACGGTAGCAACGTGAGAGGAGTACTAACTCATGGTGATCAATATGTTCAGTACAATCTGTTCGGTAACCTCTTTGAGGTCTCCAGAAAGTACGTTCCTCCCATCCGCCCTGTCGGTCGTGGAGCTTACGGTATTGTCTGTGCTGCTGTGAATTCAGAAACACGGGAAGAAGTTGCGATCAAGAAGGTTGGCAATGCTTTTGATAATAGAATAGATGCTAAGAGGACGTTACGTGAAATAAAGCTTCTATGCTACTTGCGCCATGAAAACGTGATTGCCTTATTGGACGTTATTCGACCTCCTCAGAGGGAGAACTTTAATGATGTATACATGGTTTATGACTTGATGGACACTGATCTTCACCAGATAATCCGCTCTAAGCAACAACTGAATGATGATCATTGCAGGTATTTTCTCTACCAGTTGCTGCGTGGGTTGAAGTATGTTCACTCTGCTAATGTGCTGCATCGTGACCTGAAACCAAGCAATCTCCTGCTGAATGCTAATTGTGACCTTAAAATTGGAGATTTTGGTCTTGCACGAACAACTTCTGAAGCAGATTTCATGACCGAGTATGTTGTGACTCGGTGGTATCGAGCTCCAGAACTGCTCCTAAATTGTTCAGAATATACAGCTGCAATTGATATCTGGTCCGTGGGTTGCATACTTGGAGAAATTATGACCAGAGAGCCTCTGTTTCCTGGCAAGGATTATGTTCACCAGCTTCGACTTATTACAGAGCTCATAGGTTCTCCTGATGACTCCAGCCTTGGATTTCTACGAAGCGATAATGCACGCAGGTATGTGAAGCAGCTTCCACAGTACCCAAAGCAGCATTTTCCTGCTAGATTCCCAAACGGATCTGCGATGGCTGTTGATTTGCTGGAAAAGATGCTTGTATTTGATCCTAACAAGCGAATAACAGTAAATGATGCTCTCTGCCACCCATATTTGGCCCCACTTCACGATATAAATGATGAGCCAGTCTGTTCCCAGCCTTTCAGTTTTGACTTTGAGAATCCTTCGATCACAGAGGAGCATATCAAGGAGCTAATTTGGAGGGAGTCGGTTAGATTCAACCCAGATCCTTGA |
|  |  | Protein | MTPETKSGHGAAGQNRHGSNVRGVLTHGDQYVQYNLFGNLFEVSRKYVPPIRPVGRGAYGIVCAAVNSETREEVAIKKVGNAFDNRIDAKRTLREIKLLCYLRHENVIALLDVIRPPQRENFNDVYMVYDLMDTDLHQIIRSKQQLNDDHCRYFLYQLLRGLKYVHSANVLHRDLKPSNLLLNANCDLKIGDFGLARTTSEADFMTEYVVTRWYRAPELLLNCSEYTAAIDIWSVGCILGEIMTREPLFPGKDYVHQLRLITELIGSPDDSSLGFLRSDNARRYVKQLPQYPKQHFPARFPNGSAMAVDLLEKMLVFDPNKRITVNDALCHPYLAPLHDINDEPVCSQPFSFDFENPSITEEHIKELIWRESVRFNPDP* |
| LbMAPK6 | Lb1G07414.2 | CDS | ATGACACCAGAGACGAAATCTGGCCATGGTGCTGCCGGGCAGAACCGACACGGTAGCAACGTGAGAGGAGTACTAACTCATGGTGATCAATATGTTCAGTACAATCTGTTCGGTAACCTCTTTGAGGTCTCCAGAAAGTACGTTCCTATCCGCCCTGTCGGTCGTGGAGCTTACGGTATTGTTTGTGCTGCTGTGAATTCAGAAACACGGGAAGAAGTTGCGATCAAGAAGGTTGGCAATGCTTTTGATAATAGAATAGATGCTAAGAGGACATTACGTGAAATAAAGCTTCTACGCTACTTGCGCCATGAAAACGTGATTGCCTTATTGGACGTTATTCGACCTCCTCAGAGGGAGAACTTTAATGATGTATACATGGTTTATGACTTGATGGACACTGATCTTCACCAGATAATCCGCTCTAAGCAACAACTGAATGATGATCATTGCAGGTATTTTCTCTACCAGTTGCTGCGTGGGTTGAAGTATGTTCACTCTGCTAATGTGCTGCATCGTGACCTGAAACCAAGCAATCTCCTGCTGAATGCTAATTGTGACCTTAAAATTGGAGATTTTGGTCTTGCACGAACAACTTCTGAAGCAGATTTCATGACCGAGTATGTTGTGACTCGGTGGTATCGAGCTCCAGAACTGCTCCTAAATTGTTCAGAATATACAGCTGCAATTGATATCTGGTCCGTGGGTTGCATACTTGGAGAAATTATGACCAGAGAGCCTCTGTTTCCTGGCAAGGATTATGTTCACCAGCTTCGACTTATTACAGAGCTCATAGGTTCTCCTGATGACTCCAGCCTTGGATTTCTACGAAGCGATAATGCACGCAGGTATGTGAAGCAGCTTCCACAGTACCCAAAGCAGCATTTTCCTGCTAGATTCCCAAACGGATCTGCGATGGCTGTTGATTTGCTGGAAAAGATGCTTGTATTTGATCCTAACAAGCGAATAACAGTAAATGATGCTCTCTGCCACCCATATTTGGCCCTATATCACGATATAAATGATGAGCCAGTCTGTTCCCAGCCTTTCAGTTTTGACTTTGAGAATCCTTCGATCACAGAGGAGCATATCAAGGAGCTAATTTGGAGGGAGTCGGTTAGATTCAACCCAGATCCTTGA |
|  |  | Protein | MTPETKSGHGAAGQNRHGSNVRGVLTHGDQYVQYNLFGNLFEVSRKYVPIRPVGRGAYGIVCAAVNSETREEVAIKKVGNAFDNRIDAKRTLREIKLLRYLRHENVIALLDVIRPPQRENFNDVYMVYDLMDTDLHQIIRSKQQLNDDHCRYFLYQLLRGLKYVHSANVLHRDLKPSNLLLNANCDLKIGDFGLARTTSEADFMTEYVVTRWYRAPELLLNCSEYTAAIDIWSVGCILGEIMTREPLFPGKDYVHQLRLITELIGSPDDSSLGFLRSDNARRYVKQLPQYPKQHFPARFPNGSAMAVDLLEKMLVFDPNKRITVNDALCHPYLALYHDINDEPVCSQPFSFDFENPSITEEHIKELIWRESVRFNPDP* |
| LbMAPK7 | Lb2G11155.1 | CDS | ATGATGCCGGAGACAAGCTCTGGTCATGGAGGAGCTGCATCCGATCAGCATCACCACCACCACCACAGCAGCTGCAACGTAAGAGGAGTTGTAGCCCATGGTGGACGGTATGTCCAATACAATGTCCATGGTAACTTCTTTGAAGTGTCTAGAAAGTATGTCCCACCCCTCCGCCCCGTTGGTCGTGGTGCTAGTGGTTTTGTCTGTGCCGCTGTAAATTCAGAGACACGAGAAGAGGTTGCAATCAAGAAAGTGGGGAACGCATTTGATAACATAATAGATGCTAAGAGGACCTTACGTGAAATCAAGCTTCTGCGCCATATGGATCATGGAAATGTGATTGCCATCAAAGATATAATTCGACCTCCTCTAAAAGAAAACTTCAATGATGTCTACATTGTGTATGAGTTAATGGACACCGATCTTCAACAGATCATTCGCTCCAATCAACCCCTGAATGATGAACATTGTCGGTATTTCCTCTACCAATTGCTACGAGGGTTGAAGTATGTTCACTCCGCTAATGTCCTCCACCGTGACCTGAAACCCAGCAACCTGTTACTCAACTCCAACTGTGACCTCAAGATCGGAGACTTTGGTCTAGCAAGAACAACTTCTGAGACAGATTTCATGACAGAGTATGTCGTAACTCGCTGGTACCGAGCTCCAGAGCTTCTCCTCAACTGTTCAGAGTACACTGCTGCTATTGATATCTGGTCCGTTGGTTGCATACTAGGAGAAATCATGACCCGTCATCCCCTCTTTCCTGGCAAGGATTATGTTCATCAGCTTAGACTTATCACAGAGCTCATAGGATCACCTGATGAATCCAGCCTCGGTTTCCTGCGGAGTGACAATGCAAGGAGATATGTTAGGCAGTTTCCTCGTTACCCAAAGCAGCAATTCTCAGCCAGGTTCCCGAATGCCTCTCCTCTTGCCGTTGATTTGCTGGAGAAGATGCTTGTGTTTGATCCAAACAGGCGGGTAACAGCTGCTGAAGCTCTCTGTCACCCTTACTTGGCCCCACTGCACGACCTCAACGATGAGCCAGTTTGTCCACAGCCTTTCAGTGTTGACTTTGAGCAGCCAGCTATGACGGAGGAGCACATCAAGGAGCTGATCTGGAGGGAGTCAGTTAAATTCAACCCTGATCCTTGA |
|  |  | Protein | MMPETSSGHGGAASDQHHHHHHSSCNVRGVVAHGGRYVQYNVHGNFFEVSRKYVPPLRPVGRGASGFVCAAVNSETREEVAIKKVGNAFDNIIDAKRTLREIKLLRHMDHGNVIAIKDIIRPPLKENFNDVYIVYELMDTDLQQIIRSNQPLNDEHCRYFLYQLLRGLKYVHSANVLHRDLKPSNLLLNSNCDLKIGDFGLARTTSETDFMTEYVVTRWYRAPELLLNCSEYTAAIDIWSVGCILGEIMTRHPLFPGKDYVHQLRLITELIGSPDESSLGFLRSDNARRYVRQFPRYPKQQFSARFPNASPLAVDLLEKMLVFDPNRRVTAAEALCHPYLAPLHDLNDEPVCPQPFSVDFEQPAMTEEHIKELIWRESVKFNPDP* |
| LbMAPK8 | Lb2G12330.2 | CDS | ATGGGGGGAGGAGGAGCCCTAGTCGACGGATTGCGGCGGTGGCTTGCCTGTGGAACCACCGTCGTAACCCCCGATTATTATAAATCCCACCAATTACCTCATGACTTAGACACTGTCGCGGAGGCACAGCCAGCGGAGGATATTGATCTCTCCGTTCTCAAGCCGGTTCACGTTCCCAAGCGCTATCCCTGCCACGTCGCCTTACCTGTAGATCCTCTTAAAAAGGACATGGTTGACATGGAGTTCTTCACAGAATATGGTGAAGCAAGTAGGTATGAAATTCAGGAGATGATTGGCAAGGGAAGCTATGGTGTTGTTGCTTCTGCCATTGACACTCATACTGGTGAAAAGGTGGCTATAAAGAAAATCAATGATGTCTTTGAACATGTATCAGATGCCACTCGCATTCTAAGAGAAATCAAGCTTCTTCGATTGCTTCACCACCCAGATATAGTTCAAATAAAGCATATAATGCTTCCTCCATCTCGAAGGGAATTCAAAGATATTTATGTGATGTTTGAGTTGATGGAGTCTGATCTTCACCAAGTAATAAAAGCAAACAACGATCTAACACCTGAACACCATCAATTTTTCTTGTATCAACTTCTTAGGGGACTGAAGTATATACATTCAGCAAATGTTTTTCACCGTGACTTGAAGCCAAAAAATATCCTTGCCAATGCAGACTGCAAACTGAAGATCTGTGATTTTGGACTTGCTCGTGTGTCATTTGATGATGCACCTTCTACCATCTTTTGGACAGATTATGTGGCTACACGATGGTATCGAGCACCCGAGCTTTGTGGTTCTTTCTTCTCCAAATACACTCCTGCTGTCGACATTTGGAGCATTGGATGCATATTTGCTGAATTGCTCATGGGAAAGCCTTTGTTTCCTGGAAAGAATGTTGCTCATCAGTTAGATCTTATGACCAATTTGCTTGGAACCCCTTCTCCAGAGTGCCTTTCGCGGATTCGAAATGAGAAGGCAAGAAGGTATTTGAACAATATGAAAAAGAAGGCGCCGGTCCCATTCTCACAAAAATTCCCTAAGGCTGATCCTTTGGCTCTCCGACTACTTGAGCGCTTGCTTGCCTTTGATCCTAGAGATCGTCCTTCTGCTGAAGAGGCATTGTTAGATCCATATTTTAATGGTTTAGCCCAGGTAGAAAATGAACCTACCGCACAACCAATTCCAAAGCTTGAGTTTGCATTTGAGTGGAAAAAACTAAGAAGAGACGATGTCAGAGAGCTTATATACAGGGAGATTTTAGAGTATCATCCGCAAATGTTACATGAGTATCTTCACGGGACTAATCAACCTAACTTCACATATCCAAGTGGGGTAGATCGATTCAAACGACAATTTGCCCATTTTGAGGACCATCCTGGTCAAGGAGGAAAGGGTGCTCCCTCACTTGACAGAAAGCATGTGTCCTTGCCCAGGGAGCGTGTACAAGCTGCTTTAAATGACATAGATAAGCAGAATGCGAAGGATGCATCAGAGGAGAATGCAGAAACAAATAATCGCTCGCCAGGACTCATAAAGAGCGAAAGTATCAGTGCTTCAAAGTGTGTCAAAGCGGGTAATTCAGAGGAAGGGATGACTTCTCTACAAGATGAAGTGAACAAATTATCTGGCGAGATGTCCCAATTGGATGCATAG |
|  |  | Protein | MGGGGALVDGLRRWLACGTTVVTPDYYKSHQLPHDLDTVAEAQPAEDIDLSVLKPVHVPKRYPCHVALPVDPLKKDMVDMEFFTEYGEASRYEIQEMIGKGSYGVVASAIDTHTGEKVAIKKINDVFEHVSDATRILREIKLLRLLHHPDIVQIKHIMLPPSRREFKDIYVMFELMESDLHQVIKANNDLTPEHHQFFLYQLLRGLKYIHSANVFHRDLKPKNILANADCKLKICDFGLARVSFDDAPSTIFWTDYVATRWYRAPELCGSFFSKYTPAVDIWSIGCIFAELLMGKPLFPGKNVAHQLDLMTNLLGTPSPECLSRIRNEKARRYLNNMKKKAPVPFSQKFPKADPLALRLLERLLAFDPRDRPSAEEALLDPYFNGLAQVENEPTAQPIPKLEFAFEWKKLRRDDVRELIYREILEYHPQMLHEYLHGTNQPNFTYPSGVDRFKRQFAHFEDHPGQGGKGAPSLDRKHVSLPRERVQAALNDIDKQNAKDASEENAETNNRSPGLIKSESISASKCVKAGNSEEGMTSLQDEVNKLSGEMSQLDA* |
| LbMAPK9 | Lb3G16537.1 | CDS | ATGGGGAGTGGGACAGTAGTGGATGGCGTTCGTCGCTGGTTTCAACGCCGCTCTTCATTCTCCCGCTCATCCGTCAACTCTTCTTCTTCTTCCTCTTCATTGTCAAATAGTCACAATCCAAACAGCAAAACAAGGGATAATACTTTCGAGGGCGTTGTAGATTATGAGAGTGGGACGTTGAAATATTCTAAATCTTCTGTCTCTACTGTTGATAAGGGAGATTACAGCCTAGCTAAGCAGTCGGAGGGAGGAGAAAAAGAGGATATCGAACTTGAGACAGACTTTGACCTTTGTGGATTGTCGCCCTTGAAGGTTCCGAACCGGGTTTTGGATTGCAGACACGGGTGCCGTGTTGCTCCCATGGATCCTTACAGGAAGAATAACGAGTTTTTCACAGAATATGGAGAAGCTAACCGCTACCAAGTTGAGGAGATCATAGGCAAAGGAAGCTACGGCATTGTTGGTTCAGCAATCGATTCCCAGACAGGAGAGAAAGTAGCAATAAAAAAGATCAATGATGTCTTCGAGCATGTGTCAGATGCCACCAGAATACTGAGGGAAATCAAGCTTCTGAGGCTGCTCCGTCATCCAGACATTGTGGAAATAAAGCATATAATGCTTCCCCCTTCGAGAAGAGAATTTCGGGATATTTATGTTGTCTTCGAGTTGATGGAATCTGATTTACATCAAGTGATTAAAGCAAATGATGATCTAACTCCAGAGCATCATCAGTTTTTTTCTAATGTCTTCCATCGAGATTTGAAGCCGAAAAATATCCTTGCTAATGCTGATTGCAAGTTGAAAATTTGTGATTTTGGGCTTGCTCGAGTGTCTTTTAATGATGCCCCTTCTGCTATCTTTTGGACTGATTATGTAGCAACACGATGGTATCGAGCTCCTGAACTTTGTGGATCATTTTTCTCGAAGTATACGCCAGCAATTGATATTTGGAGTATAGGGTGCATCTTCGCCGAAATGCTATTGGGAAAGCCATTATTTCCAGGGAAAAACGTGGTGCATCAACTGGATCTAATGACGGATTTTCTTGGAACTCCATCGCTGGAAGCCATTCAAAGAATCAGAAATGAAAAGGCTAAACGATACCTTGCTGGCATGAAGAAGAAACAACCAGTTCCATTCTCCCGGAAGTTCCCTCATGCAGATCCATTAGCTCTTGGACTGCTAGAACGTCTTCTTGCTTTTGATCCTAGAGACCGCCCATCGGCCGAACAGGCTCTGGCTCATCCGTACTTCAATGGATTGGCAAATGTCGAACGAGAACCATCTACCCAGCCCATCTCAAAATTGGAATTTGAATTTGAGAGAAGGAAGCTGGCGAAAGATGATGTTAGGGAACTAATTTACCGGGAGATATTAGAGTATCATCCTCAGATGCTTCAGGAGTATCTTCGTGGAGGGGATCAGACCAGCTTCATGTATCCTAGTGGCGTGGATCGATTCAAGCGACAGTTTGCACACCTTGAAGAGAATTTTGGTAAAGGAGAAAGAGGAAGTACCCCTCCTCTTCAGAGACAACATGCTTCCCTTCCAAGACAACGTGTACCCGAGGCTGCGGATCAATTATCAAAAGAAGATGAAGAATTAGAAAAGAGAAGCGCTGCTGCTGCTGCAAAAACAGCTCTTGAAAGTCCAGAAGGTGATCAGCAGCCAAATGGTAACAGTAAACCCAACGCTTCTGCTCGAAGCCTTATGAAAAGCGCTAGCATTAGTTCGAGATGTATCGGTCCGCATTCACAGAGAGCTCCAGAGGATGATCCTATATACGAGTTGGATGAAAATGATCGATGGTCTGACTGA |
|  |  | Protein | MGSGTVVDGVRRWFQRRSSFSRSSVNSSSSSSSLSNSHNPNSKTRDNTFEGVVDYESGTLKYSKSSVSTVDKGDYSLAKQSEGGEKEDIELETDFDLCGLSPLKVPNRVLDCRHGCRVAPMDPYRKNNEFFTEYGEANRYQVEEIIGKGSYGIVGSAIDSQTGEKVAIKKINDVFEHVSDATRILREIKLLRLLRHPDIVEIKHIMLPPSRREFRDIYVVFELMESDLHQVIKANDDLTPEHHQFFSNVFHRDLKPKNILANADCKLKICDFGLARVSFNDAPSAIFWTDYVATRWYRAPELCGSFFSKYTPAIDIWSIGCIFAEMLLGKPLFPGKNVVHQLDLMTDFLGTPSLEAIQRIRNEKAKRYLAGMKKKQPVPFSRKFPHADPLALGLLERLLAFDPRDRPSAEQALAHPYFNGLANVEREPSTQPISKLEFEFERRKLAKDDVRELIYREILEYHPQMLQEYLRGGDQTSFMYPSGVDRFKRQFAHLEENFGKGERGSTPPLQRQHASLPRQRVPEAADQLSKEDEELEKRSAAAAAKTALESPEGDQQPNGNSKPNASARSLMKSASISSRCIGPHSQRAPEDDPIYELDENDRWSD* |
| LbMAPK10 | Lb4G23192.1 | CDS | ATGCAACAGGATCAGAGAAGAAAGATCTCCAAGGAAGCTGATTTCTTCACCGAATATGGTGATGGCAACAGATACAAGATTCTTGAAGTTATTGGGAAGGGAAGTTATGGGGTTGTCTGTGCAGCAATTGATACGCACACAGGTGAAAAAGTAGCCATAAAGAAGATAACCGATATTTTTGAGCACACCTCAGATGCTATTAGAATCCTTCGTGAGGTCAAGTTGCTTAGGCTATTGAGGCATCCTGATATTGTTGAGATTAAGCGCATCATGTTGCCACCTTCACGGAGGGACTTCAAGGATATTTATGTTGTTTTTGAGCTTATGGAATCTGATCTTCACCAAGTCATCAAGGCAAATAATGATCTGACTCGCGAACACCACCAGTTCTTTCTTTATCAAATGCTTCGAGCTTTGAAGTATATGCACACAGCAAATGTTTACCATCGAGATCTTAAACCAAAGAATATCTTGGCTAATGCAAACTGCAAGCTAAAAATCTGTGATTTTGGGCTTGCTAGAGTGGCATTTAGCGATACTCCTACATCAGTATTCTGGACTGATTACGTTGCCACTAGGTGGTACAGGGCTCCAGAGCTTTGCGGATCATTTTATGCAAAGTATACATCAGCGATTGATATGTGGAGCATTGGATGCATCTTTGCAGAGGTATTAACCGGGAAGCCACTATTCCCTGGGAAAAGTGTGGTCCATCAGTTGGATTTGATTACTGATCTTCTAGGGACACCCTCACGTGAGGCCATTTCATTGGTTCGTAATGATAAGTCACGTAAATACTTGACTGATATGAGGAGGAAGAATCCTGTCCCGTTCGCCCAGAAATTCCCAAAAGTAGATCCCTTGGCTTTGCGCCTTCTTGAAAGAATGTTAGCATTTGACCCCAAGGACCGTCCATCTGCGGAGGAGGCACTTGCTGATCCATACTTCAAGGGCCTGGCTAAAATGGAGAGAGAACCTTCTAGCCAACCTATCTCAAAGCTTGAATTTGACTTTGAGAGAAGAAGGTTGACCAAAGAAGACATCAGGGAATTGATTTACCGTGAGATATTGGAGTATCATCCACAGTTGCTTAAAGACTACATTAATGGAAATGAAGGGGCGAGTTTTATTTATCCCAGCGCTCTTGGTCAATTCAAAAGGCAGTTTGCTCACTTGGAGGAAAACTACGGCAGGAGTGGCCCTGTCATTCCATTGGAGAGAAAACATCTCTCTGTTCCAAGGTCCACAGTGCATTCTAGCACCATTCCACCCAGACCACAGCCTAGTATCCCACCATGCCAGCAGAGTAAAGAAGAGGGCAGGGTTCTTGTAGGTTTAAATTCTGGGAGACAGCCAGCTAGGGTTCCAGTTGGGACACCAGTAAGGGTAGTGGGTCCAGTCACGGTCTCGTATGAAATTGGCCGATCCATTCCCATGCCCCAAGAGAGTTGTGAAGCAAGGATTGTTGTTCAACAGCATGCTGTTCTCCCCCCTCAAGGGTTTACCACACCCCAATATTTCATCCGCTCTACAAGCACACCACCTCCACATCATCAAACCAAACCTACCCATTGTTCCGAGGGCCTGATGTTGAGGCCTGCAGCAGCACCAGTCAAGCAGCAGGTTCCAATGAACAATGGTGGAGCCATCATGATCACGAACCAACAGCATAGCTATAGCTGTAGCCACACACATGAGATCGCCATTGATATTAATGCTGCCAGTCACTACTATCAACCAGTGCGCCACTCCCCCATTCAACAGAAGCATATTGACCCGTATAATAGCAGAGTGGCTTTTGATGCGAAGATAATGCAAGCTCAGACCCAGTTTGGGGCTGCTGCTGTTGTCATGGGTACACACAGGAATGTGGGGACAGTTCAGTATGGCATGACCTAG |
|  |  | Protein | MQQDQRRKISKEADFFTEYGDGNRYKILEVIGKGSYGVVCAAIDTHTGEKVAIKKITDIFEHTSDAIRILREVKLLRLLRHPDIVEIKRIMLPPSRRDFKDIYVVFELMESDLHQVIKANNDLTREHHQFFLYQMLRALKYMHTANVYHRDLKPKNILANANCKLKICDFGLARVAFSDTPTSVFWTDYVATRWYRAPELCGSFYAKYTSAIDMWSIGCIFAEVLTGKPLFPGKSVVHQLDLITDLLGTPSREAISLVRNDKSRKYLTDMRRKNPVPFAQKFPKVDPLALRLLERMLAFDPKDRPSAEEALADPYFKGLAKMEREPSSQPISKLEFDFERRRLTKEDIRELIYREILEYHPQLLKDYINGNEGASFIYPSALGQFKRQFAHLEENYGRSGPVIPLERKHLSVPRSTVHSSTIPPRPQPSIPPCQQSKEEGRVLVGLNSGRQPARVPVGTPVRVVGPVTVSYEIGRSIPMPQESCEARIVVQQHAVLPPQGFTTPQYFIRSTSTPPPHHQTKPTHCSEGLMLRPAAAPVKQQVPMNNGGAIMITNQQHSYSCSHTHEIAIDINAASHYYQPVRHSPIQQKHIDPYNSRVAFDAKIMQAQTQFGAAAVVMGTHRNVGTVQYGMT* |
| LbMAPK11 | Lb4G24221.1 | CDS | ATGCAAAGAAAGGGGTCTGTTGATCTGGATTTCTTCACCGAATATGGTGAGGGTAGTAGGTATAAGGTTGAGGAAGTGATTGGTAAAGGAAGTTACGGTGTTGTTTGCTCCGCGATTGATACCCACCTTGGAGAGAAAGTTGCAATCAAGAAGATCAATGATATCTTCGAGCATGTCTCTGATGCTACACGCATACTTAGAGAGATCAAGCTTCTTCGGCTATTGAGACACCCTGATATTGTGGAGATCAAACACATTTTGTTGCCTCCCTCTAGAAGGGAGTTTAAGGACATATATGTTGTCTTTGAGCTCATGGAATCTGATCTGCATCAGCCCAAAAACATCCTAGCAAATGCTGATTGTAAGCTCAAGATTTGTGACTTTGGTCTTGCACGAGTGGCTTTCAATGATACCCCAACTGCTATATTCTGGACGGACTATGTTGCAACAAGATGGTATAGAGCTCCTGAATTGTGTGGATCATTTTTCTCCAAGTATACACCAGCGATAGACATATGGAGCATTGGATGCATATTTGCTGAACTTCTTACCGGGAAACCTCTTTTCCCAGGCAAAAATGTAGTGCACCAGCTTGATCTGATGACAGATTTGCTTGGAACACCTTCACCTGAAGCCATAGCCAGAATACGTAATGAGAAGGCTAGGAGGTACTTAAGCAGCATGCGCAAGAAGAAACCTATCCCTTTCACACACAAATTTCCAAATGCTGATCCTCTAGCTCTTCGTTTGCTGGAAAGAATGTTGGCATTTGAGCCCAAGGACAGGCCTAATGCCGAGGAGGCTCTTGCAGATCCATACTTCAGAGGCTTGGCCAAGGTTGAGAGAGAACCTTCGGCTCAACCTGTTACCAAGATGGAATTTGAATTCGAGAGGAGAAGGGTGACAAAGGAAGATGTTAGGGAGCTAATATATCGTGAAATTTTGGAGTATCATCCAAAGATGTTGAAAGAACACTTGGAAGGAGAGGAGCCAACGGGCTTCATGTACCCGAGTGCTGTTGATCACTTCAAGAAGCAATTCGCTTTCCTCGAGGAGCACTACAAAAATGGCTCAACTGGAACCCCGCCAGAGAGGCAACATGCTTCATTACCAAGGGCATGTGTACAGTACTCTGACAACTCAATGCAGACTTCAGCTGAAGTTACTGATGATCTCTCAAAATGCCGTATCAAGGATTCTGAGAAAGCATATGTCCACAGGAATGGTGATCCAGTCCCTATGGCTAGGCTTCCTCAAAATGTACAAGTTGAAGGGGTGGCTGCGAGGCCTGGGAAGGTGGTTGGTTCTTTGCTTCGGTACAACAACTGTGGCGCAGCTGCGAATGCAGAGGTGGTTAATGACCAGCGAAGAATGGGGAGGAACCTTAACGGTGTGCCATCACAATTTGGAGGACCCATTAGTGCATACCCTAAGAGAACTCCTGTTTGTAAAAATGAAAGAGGAGAAGATAGTGGCAGTGGGGTTGTTGAAGGGTCAAATGGGCTCCAACCTAAGCCCTCACCATACATGCCCAGAAAGATGGCTGCTGCCCCAAATGGAACTGCTAATCATTGGTATTGA |
|  |  | Protein | MQRKGSVDLDFFTEYGEGSRYKVEEVIGKGSYGVVCSAIDTHLGEKVAIKKINDIFEHVSDATRILREIKLLRLLRHPDIVEIKHILLPPSRREFKDIYVVFELMESDLHQPKNILANADCKLKICDFGLARVAFNDTPTAIFWTDYVATRWYRAPELCGSFFSKYTPAIDIWSIGCIFAELLTGKPLFPGKNVVHQLDLMTDLLGTPSPEAIARIRNEKARRYLSSMRKKKPIPFTHKFPNADPLALRLLERMLAFEPKDRPNAEEALADPYFRGLAKVEREPSAQPVTKMEFEFERRRVTKEDVRELIYREILEYHPKMLKEHLEGEEPTGFMYPSAVDHFKKQFAFLEEHYKNGSTGTPPERQHASLPRACVQYSDNSMQTSAEVTDDLSKCRIKDSEKAYVHRNGDPVPMARLPQNVQVEGVAARPGKVVGSLLRYNNCGAAANAEVVNDQRRMGRNLNGVPSQFGGPISAYPKRTPVCKNERGEDSGSGVVEGSNGLQPKPSPYMPRKMAAAPNGTANHWY* |
| LbMAPK12 | Lb5G27787.1 | CDS | ATGGAGAAGCTTGATAAACAACGTCGTAAAGAGGGCTTGGTTAAACTCTTCCCAAAAAAGTGCTTTAAGAGCTTACAATTTTGCTATGGCTCTTTCCACAAGGATTCTCATACACAGGCTGAATCTGATCTGCAGCAACTAGAACTAGGGCAGCAGCAGGAGTTGGAGCAGAATGCAATTATGTCAACAGAAAGACATAGTAAACAACATCAAGGGGGTTTGCTTAAACTCTTTTCAAAGAAGTGCTCCAAGAGCTTGAAATTATGTGGCTATAAGTGTTCTCATACTCTAACCAAGTCCAAGAAGCAGGAAACGCAACAACAGATGTTGCGATTGATGTATGACTATGAGAATTTGAAAGAGGAAAATCTGGTGATATCTCATGAGCTGGAGGACAAAACCCAACTTCATAAACTGCTAGATCTTCATTACAAAAGCTTGAGTTCATATGCTACTATAAAAGAAGTCGAGGTAGATAAACTTGAGAGAGAGGTCAAGATTTGGACTACAAGAGCGACCAAGGCAGAAACAGCCCTTGAAGTCAAAGATAAGGTTACCTCCCGAGCATTAGCCGAAGCTGCCGAATTAAGGCTGGTGAATGACAATCTCAAGAAAGATCATGAAGAGACAATGAAACAAGTTCAAACACTGAAAGAAGGATATGAATTTCTAGTAGTTGATTTGATCAGTCAGGTTCAACAAGGAAACATATTAAAGACTGGGTTAATGGAAGAGTTGATGGAAGTAAAGTCTCACAATGATAAGAAGGAAATGACTCTGAAAAGTTTGAGTTCAGAAGTGGAAATATTAAAACAACAAAATGTAGAACTGAGAAGTTTGGTCACAAGGGGTGACTCAGAAAAGAAGAAGTTAGAGAGTGAAGTTGACCAACTCAGAACCCATCTTGTCAGCTTAACAAACATTGCGAAATCATCACTTGAGAACCTGAAGATGACAAGATCTATGAATGGAGAAGACTTTGCTCTGGAAAAGTTAAAGTTGGAAGTAGAAGGACTAAGAATTCAAAATTCGGATCTGTGCAAGTTGGTGTATCAACATGAGTTGGAGAAAAAGATATCGAGCAATCTAGTGTTGCAGATCAATAGTGAGAACTCCACCATACAACAGATTGCAGAATTATTGGAGCCACCTGTGTCAGTATTGCAGCCCTTCATGATCAACATGCATCCTCCTCCTGGATTTGAGGCACAAGCAGAGAAGAAGCCTTGCAAAGAAGGAATGATAGAATTTCGAAAGACTTCTTTTCTCATCTCAAACACACTGTTTGAAGTGGATGCTAAGTATTCCCCAATGAAGCTGCTGGGAAGTGGGTCATATGGAGTGGTATGCTCCTTCATGGATAAAGAAACTGGTGAAATGGTTGCCATTAAGAAGATTCAGGATAGCTTCTTCTGTCAAGTGGCGGGTTTGAGGACACTAAGAGAAATACGGATTCTAAGACATCTTAATCATCCAAATGTAATCACCTTGAAAGATGTCATGCTCCCAGTTGATAGGTCAACTTTCAAGGAAGCTTACCTTGTATACGAGTTAATGGATTCTGACCTTGAGCAGGTTATTAGTTCGCCCATGCACCTCACAGTTGATCACTATAGACATATTATGCTTCAGATACTATTAGGGCTAAAGTATCTGCATAACGGGAACATTCTTCACCGAGACCTCAAGCCAGCCAATCTATTCATAGATAAAAACTTTAACATCAAGATTGGTGACTTCGGGCTAGCCAGAACAACAACATATGCAACCCAAGGCCAAGAAATTGATTGGCATTTCACTGATTATGTTGTCTCAAGGTGGTACCGAGCTCCAGAGATTCTCCTTGGCTGCCCCTATGGGAAGCCAGCAGATATGTGGTCAGTAGGGTGCATCTTTGCGGAGATCCTCGCTCGAAAGCCCTTCTTGCCTGGAGCTGATAGCCAAGACCAGTTAGACTGGATTCTCTCCCTTGTTGGGAGGCATATGAAGGATGATTTGAAGTTTATTCACTCCGAAAATTGCAAGCAATATATTGTTGACCGGTCATGTGACCTGGGGTCTGGAGTTGACTTATTTCAGCTATTTCCGGAGTCTGAGAGGCTGGCTATTGATCTTCTCAGAAGATTTCTCACTTTTAACCCAGCTGAGAGGATTACTATTGCTGAAGCATTGCATCATCCTTATTTTTGTGAGATATATGACCCGATGGTTATCCTGCCAGAACTAGAGCCCATTGACGTTGAAGTGAAGGGCAATGTGACGAATGTGTCTATATTGAGAGAATTGGTCTGGGAGGAGATGCTGCATTTTAATGCTGCTGTTTCGACCTAG |
|  |  | Protein | MEKLDKQRRKEGLVKLFPKKCFKSLQFCYGSFHKDSHTQAESDLQQLELGQQQELEQNAIMSTERHSKQHQGGLLKLFSKKCSKSLKLCGYKCSHTLTKSKKQETQQQMLRLMYDYENLKEENLVISHELEDKTQLHKLLDLHYKSLSSYATIKEVEVDKLEREVKIWTTRATKAETALEVKDKVTSRALAEAAELRLVNDNLKKDHEETMKQVQTLKEGYEFLVVDLISQVQQGNILKTGLMEELMEVKSHNDKKEMTLKSLSSEVEILKQQNVELRSLVTRGDSEKKKLESEVDQLRTHLVSLTNIAKSSLENLKMTRSMNGEDFALEKLKLEVEGLRIQNSDLCKLVYQHELEKKISSNLVLQINSENSTIQQIAELLEPPVSVLQPFMINMHPPPGFEAQAEKKPCKEGMIEFRKTSFLISNTLFEVDAKYSPMKLLGSGSYGVVCSFMDKETGEMVAIKKIQDSFFCQVAGLRTLREIRILRHLNHPNVITLKDVMLPVDRSTFKEAYLVYELMDSDLEQVISSPMHLTVDHYRHIMLQILLGLKYLHNGNILHRDLKPANLFIDKNFNIKIGDFGLARTTTYATQGQEIDWHFTDYVVSRWYRAPEILLGCPYGKPADMWSVGCIFAEILARKPFLPGADSQDQLDWILSLVGRHMKDDLKFIHSENCKQYIVDRSCDLGSGVDLFQLFPESERLAIDLLRRFLTFNPAERITIAEALHHPYFCEIYDPMVILPELEPIDVEVKGNVTNVSILRELVWEEMLHFNAAVST* |
| LbMAPK13 | Lb5G27798.1 | CDS | ATGGTGGTAAAGTCTCATAATGATAAGAAGGAAATGGCTCTGAAAAGTTTGAGTTCAGAAGTGGAAATATTAAAACAACAGAATGTAGAGTTGAGAAGTTTGGTTCATAAGGGTGAACCAGAAAAGAAGAAGTTAGAGAGTGAAGTTGACCAACTCAGAACCCATCTTGTCAGCTTAACAGACATTGCGAAATCATCACTTGAGCACAAGGAAGCACTCATGGTTGAGTTGATTGAGAACCTGAAGATGACAAGATCTATGAATGACGAGAAGACTTTGGCTCTGGAAAAGTTAAAGTTGGAAGTAGAAGGACTAAGACTTCAGAATTTGGATCTGAGCAAGTTGGTGTATCACCATGAAATGGAGAAACCCACCATACCACAGATTGCAGAATCATTTGAGCCACCTATGTCAGTATTGCAGCCCTCCATGATCAACATGCATCCTCCTCCTGGATTTGAGGCACGAGCAGAGAAGAAGCTTTGCAAAGAAGGAATAAGAGAATTTCGAAAGACTTCTTTTCTCATCTCAAACACACTGTTTGAAGTGGATGCTAAGTATTCCCCAATGAAGTTGCTGGGAAGTGGATCATATGGAGTGGTATGCTCCTTCATGGATAAAGAAATTGGTGAAATGGTTGCCATTAAGAAGATTCAGAATGCCTTCTTCTGTCAAGTGGCGGGTTTGAGGACACTAAGAGAAATACGGATTCTAAGACATCTGAATCATCCTAATGTAATCACCTTGAAAGATGTCATGCTCCCAACTGATAGGTCAACTTTCAAGGAAGCTTACCTTGTATACGAGTTGATGGATTGTGACCTTGATCAGGTTATTAGTTCAACCATGCACCTCACAGCTGACCACTGTAGACATATTATGCTTCAGATACTATTAGGGCTAAAGTATCTGCATAACGGGAACATTCTTCACAGAGACCTCAAGCCAGCCAATCTATTCATAGACAAAAACTTCAACATCAAGATTGGTGACTTCGGGCTAGCCAGAACAACAACATATGGAACCCAAGACCAAGAAATTGATTGGCATTTCACTGATTATGTTGTCTCAAGGTGGTACCGAGCTCCAGAGATTCTCCTCGGCTGCCCCTATGGGAAGTCAGCTGATATGTGGTCAGTAGGGTGCATCTTTGCGGAGTTCCTTGCTCGAAAGCCCTTATTGCCTGGAGCTGACAGCCAAGACCAGTTAAACTGGATCCTTTCCCTTGTTGGGAGGCAGACGAAGGATGATTTGGAGTTTGTTCACTCTGAACAATGTAAGCAATATATTGTTGACCGATCATGGGACTTGGGGTCTGGAGTTGACTTATTTCAGCTATTTTCAAAGTCTGAGAGGCTGGCTATTGATCTCCTAAGAAGAATGCTCATTTTCAACCCAACTGAGAGGATTACTGTTGCTGAAGCATTGCATCATCCTTTCTTTTGTGAGATATATGACCCGATGGTTCTTCTGCCAGAACTAGAGCCCATTGACGTTGAAGTGAAGGGCAATATGCTGGATGTATCTATATTGAGAGAATTGGTATGGGAGGAGATGCTGCATTTTAATGTTGTTGGTTTTAGCTAA |
|  |  | Protein | MVVKSHNDKKEMALKSLSSEVEILKQQNVELRSLVHKGEPEKKKLESEVDQLRTHLVSLTDIAKSSLEHKEALMVELIENLKMTRSMNDEKTLALEKLKLEVEGLRLQNLDLSKLVYHHEMEKPTIPQIAESFEPPMSVLQPSMINMHPPPGFEARAEKKLCKEGIREFRKTSFLISNTLFEVDAKYSPMKLLGSGSYGVVCSFMDKEIGEMVAIKKIQNAFFCQVAGLRTLREIRILRHLNHPNVITLKDVMLPTDRSTFKEAYLVYELMDCDLDQVISSTMHLTADHCRHIMLQILLGLKYLHNGNILHRDLKPANLFIDKNFNIKIGDFGLARTTTYGTQDQEIDWHFTDYVVSRWYRAPEILLGCPYGKSADMWSVGCIFAEFLARKPLLPGADSQDQLNWILSLVGRQTKDDLEFVHSEQCKQYIVDRSWDLGSGVDLFQLFSKSERLAIDLLRRMLIFNPTERITVAEALHHPFFCEIYDPMVLLPELEPIDVEVKGNMLDVSILRELVWEEMLHFNVVGFS* |
| LbMAPK14 | Lb5G29170.1 | CDS | ATGCAAAGAAAGGGGTCTGTTGATCTGGATTTCTTCACCGAATATGGTGAGGGTAGTAGGTATAAGGTTGAGGAAGTGATTGGTAAAGGAAGTTACGGTGTTGTTTGCTCCGCGATTGATACCCACCTTGGAGAGAAAGTTGCAATCAAGAAGATCAATGATATCTTCGAGCATGTCTCTGATGCTACACGCATACTTAGAGAGATCAAGCTTCTTCGGCTATTGAGACACCCTGATATTGTGGAGATCAAACACATTTTGTTGCCTCCCTCTAGAAGGGAGTTTAAGGACATATATGTTGTCTTTGAGCTCATGGAATCTGATCTGCATCAGGTTATCAAAGCAAATGATGATTTGACTCCAGAGCATTATCAGTTTTTTTCTATCACAAATGTTTTCCATCGGGACCTGAAGCCCAAAAACATCCTAGCAAATGCTGATTGTAAGCTCAAGATTTGTGACTTTGGTCTTGCACGAGTGGCTTTCAATGATACCCCAACTGCTATATTCTGGACGGACTATGTTGCAACAAGATGGTATAGAGCTCCTGAATTGTGTGGATCATTTTTCTCCAAGTATACACCAGCGATAGACATATGGAGCATTGGATGCATATTTGCTGAACTTCTTACCGGGAAACCTCTTTTCCCAGGCAAAAATGTAGTGCACCAGCTTGATCTGATGACAGATTTGCTTGGAACACCTTCACCTGAAGCCATAGCCAGAATACGTAATGAGAAGGCTAGGAGGTACTTAAGCAGCATGCGCAAGAAGAAACCTATCCCTTTCACACACAAATTTCCAAATGCTGATCCTCTAGCTCTTCGTTTGCTGGAAAGAATGTTGGCATTTGAGCCCAAGGACAGGCCTAATGCCGAGGAGGCTCTTGCAGATCCATACTTCAGAGGCTTGGCCAAGGTTGAGAGAGAACCTTCGGCTCAACCTGTTACCAAGATGGAATTTGAATTCGAGAGGAGAAGGGTGACAAAGGAAGATGTTAGGGAGCTAATATATCGTGAAATTTTGGAGTATCATCCAAAGATGTTGAAAGAACACTTGGAAGGAGAGGAGCCAACGGGCTTCATGTACCCGAGTGCTGTTGATCACTTCAAGAAGCAATTCGCTTTCCTCGAGGAGCACTACAAAAATGGCTCAACTGGAACCCCGCCAGAGAGGCAACATGCTTCATTACCAAGGGCATGTGTACAGTACTCTGACAACTCAATGCAGACTTCAGCTGAAGTTACTGATGATCTCTCAAAATGCCGTATCAAGGATTCTGAGAAAGCATATGTCCACAGGAATGGTGATCCAGTCCCTATGGCTAGGCTTCCTCAAAATGTACAAGTTGAAGGGGTGGCTGCGAGGCCTGGGAAGGTGGTTGGTTCTTTGCTTCGGTACAACAACTGTGGCGCAGCTGCGAATGCAGAGGTGGTTAATGACCAGCGAAGAATGGGGAGGAACCTTAACGGTGTGCCATCACAATTTGGAGGACCCATTAGTGCATACCCTAAGAGAACTCCTGTTTGTAAAAATGAAAGAGGAGAAGATAGTGGCAGTGGGGTTGTTGAAGGGTCAAATGGGCTCCAACCTAAGCCCTCACCATACATGCCCAGAAAGATGGCTGCTGCCCCAAATGGAACTGCTAATCATTGGTATTGA |
|  |  | Protein | MQRKGSVDLDFFTEYGEGSRYKVEEVIGKGSYGVVCSAIDTHLGEKVAIKKINDIFEHVSDATRILREIKLLRLLRHPDIVEIKHILLPPSRREFKDIYVVFELMESDLHQVIKANDDLTPEHYQFFSITNVFHRDLKPKNILANADCKLKICDFGLARVAFNDTPTAIFWTDYVATRWYRAPELCGSFFSKYTPAIDIWSIGCIFAELLTGKPLFPGKNVVHQLDLMTDLLGTPSPEAIARIRNEKARRYLSSMRKKKPIPFTHKFPNADPLALRLLERMLAFEPKDRPNAEEALADPYFRGLAKVEREPSAQPVTKMEFEFERRRVTKEDVRELIYREILEYHPKMLKEHLEGEEPTGFMYPSAVDHFKKQFAFLEEHYKNGSTGTPPERQHASLPRACVQYSDNSMQTSAEVTDDLSKCRIKDSEKAYVHRNGDPVPMARLPQNVQVEGVAARPGKVVGSLLRYNNCGAAANAEVVNDQRRMGRNLNGVPSQFGGPISAYPKRTPVCKNERGEDSGSGVVEGSNGLQPKPSPYMPRKMAAAPNGTANHWY* |
| LbMAPK15 | Lb6G31696.1 | CDS | ATGTCTACATTGCCACAGAGCTCATGGACACTGATCTTCATCAGATCATTCGGTCCAACCAGCCTTTATCTGAGGAGCATTCTCAGTACTTCTTGTACCAGATTTCGGGGGCTGAAGTACATCCACTCAGCAAATGTGCTTCATAGAGATCTAAAGCCAAGCAACCTCTTGATAAACGCAAACTGTGATCTGAAGATTTGTGACTTTGGCCTTGCGCGGCCAACTTGTGAGAATGAACACATGACTGAATATGTTGTCACCAGATGGTACAGAGCACCTGAGCTATTGCTCAACTCTTCCGACTATACAGCTGCAATTGATGTCTGGTCTGTTGGGTGTATCTTCATGGAACTTATGAACAGAAAGCCTCTCTTTCCAGGGAGAGACCATGTCCACCAAATGCGCTTATTGACAGAGCTACTTGGAACTCCAACCGAATCTGATCTTCGTTTCCTTCAGAACGAAGATGCCAGGAGGTACATTAGGCAGATGACACCTATTCCTCGCCAGCCACTAGGAAGGATGTTTCCTCACATTAATCCGTTAGCAATTGATCTGGTTGAGAGGATGCTGACATTTGATCCTACTCGAAGAATAACTGTTGAAGAGTCGTTATCTCATCCTTACTTAGCAAGACTACATGATGTTGATGATGAACCAAAATGCTCAGAGCTGTTCTCCTTTGAGTTTGAGCGTCAAGCTCTTGGAGAGGAAGAGATGAAGGAAATGATCTATAGAGAGACATTGGCTCACAACCCAGGGTTTGTATAA |
|  |  | Protein | MSTLPQSSWTLIFIRSFGPTSLYLRSILSTSCTRFRGLKYIHSANVLHRDLKPSNLLINANCDLKICDFGLARPTCENEHMTEYVVTRWYRAPELLLNSSDYTAAIDVWSVGCIFMELMNRKPLFPGRDHVHQMRLLTELLGTPTESDLRFLQNEDARRYIRQMTPIPRQPLGRMFPHINPLAIDLVERMLTFDPTRRITVEESLSHPYLARLHDVDDEPKCSELFSFEFERQALGEEEMKEMIYRETLAHNPGFV* |
| LbMAPK16 | Lb7G33589.1 | CDS | ATGGACGTTGCCCCACAAAGCTCCGACGCCGAGATGGCGGATGCGGCAGCTGAACAGCAAGCCCAACCTCCGTCTCAGCAACCGCCGCATCCTGGTGCGGGTCAAGATCCTTCCCAACATCAACATCCGCAACAGCAGATAAATATCCCTTCAACTCTTAGCCACGGTGGGCGTTTTATCCAATATAATATTTTCGGCAACATCTTTGAGGTCACTGCCAAGTACAAACCTCCTATCATGCCAATCGGCAAAGGTGCATATGGCATTGTTTGTTCGGCCTTGAATTCAGAGACTAATGAGCAGGTGGCGGTGAAGAAGATCGCGAATGCTTTTGACAATAAAGTGGATGCTAAAAGAACACTTCGCGAGATCAAACTTCTACGACACATGGACCATGAAAATGTGGTTGCAATCAGAGATATTATACCTCCTCCGAGAAGAGAGGCTTTCAATGATGTGTATATTGCATATGAACTAATGGACACAGATCTTCATCAAATTATCCGCTCCAATCAAGCTTTATCAGAGGAGCATTGCCAGTATTTTCTCTACCAAATTCTTCGTGGATTGAAGTACATACACTCAGCAAATGTTCTCCACAGGGACTTGAAGCCCAGCAACTTGCTACTAAATGCTAATTGTGATCTAAAAATATGTGATTTTGGACTTGCCCGTGTGACATCCGAAACTGATTTTATGACTGAATATGTTGTTACGAGATGGTATCGTGCACCAGAGCTGTTGTTGAACTCTTCAGATTACACTGCAGCTATTGACATATGGTCTGTTGGCTGCATATTTATGGAACTAATGGATCGCAAGCCTCTGTTCCCTGCTGATTGGCACTCCTTCAGAGCTGAGTTGGGGTTTTTAAATGATAATGCTAAAAGATATATTCGGCAACTTCCTATGCACAGAAGGCAGTCGCTCCTGGAAAAGTTCCCACATGTGCACCCATCAGCCATTGATCTTATTGAGAAGATGTTGACGTTTGATCCTAGGCAACGAATAACAGTTGAAGACGCGCTTGCACATCCATACCTGAACTCATTGCATGATATAAGTGATGAGCCAGTATGCACGACTCCATTCAACTTTGATTTCGAGCAGCATGCTTTGACAGAGGAGCAGATGAGGGAGCTCATATACCGGGAGGCACTTGCTTTCAACCCTGAGTATCAACAGTGA |
|  |  | Protein | MDVAPQSSDAEMADAAAEQQAQPPSQQPPHPGAGQDPSQHQHPQQQINIPSTLSHGGRFIQYNIFGNIFEVTAKYKPPIMPIGKGAYGIVCSALNSETNEQVAVKKIANAFDNKVDAKRTLREIKLLRHMDHENVVAIRDIIPPPRREAFNDVYIAYELMDTDLHQIIRSNQALSEEHCQYFLYQILRGLKYIHSANVLHRDLKPSNLLLNANCDLKICDFGLARVTSETDFMTEYVVTRWYRAPELLLNSSDYTAAIDIWSVGCIFMELMDRKPLFPADWHSFRAELGFLNDNAKRYIRQLPMHRRQSLLEKFPHVHPSAIDLIEKMLTFDPRQRITVEDALAHPYLNSLHDISDEPVCTTPFNFDFEQHALTEEQMRELIYREALAFNPEYQQ* |
| LbMAPK17 | Lb7G34942.1 | CDS | ATGCAGCAAGATCAGAGAAAGAAGAGCCTCAAGGATGCAGAATTCTTCACCGATTATGGTGATGCCAATAGATACAAACTTCTTGAAGTTATTGGGAAGGGGAGTTATGGAGTTGTCTGTGCAGCTATTGACACACACACTGGGGAGAAAGTGGCCATAAAGAAGATTAATGATATTTTCGAGCACAACTCTGATGCTATCCGGATCCTTCGAGAGGTTAAGTTGCTGAGGCTGTTGCGGCATCCTGATATTGTTGAAATCAAACGCATAATGATGCCGCCTACGAGGAGAGACTTCAAAGATATATATGTAGTTTTTGAGCTTATGGAGTCTGATCTTCACCAAGTCATCAAAGCTAATGATGACTTGACACGTGAGCACCATCAGTTCTTTCTGTACCAAATGCTGCGAGCGTTGAAATATATGCACACAGCAAATGTTTATCATCGAGATCTCAAACCTAAGAATATATTGGCCAATGCCAATTGCAAATTGAAAATCTGTGACTTTGGGCTTGCCAGAGTAGCATTCACTGACGCCCCTACAACAGTATTTTGGACTGATTATGTTGCTACGAGATGGTATCGGGCTCCGGAGCTGTGTGGATCATTTTCCTCCAAGTATACACCAGCGATTGATATGTGGAGTATAGGATGCATCTTCGCAGAGGTATTAACTGGAAAGCCACTTTTCCCTGGGAAAAGTGTTGTTCATCAGCTAGATTTGATCACAGATCTCCTTGGAAAACCCTCACAAGAAGTGATATCGGGGGTTCGCAATGACAAGGCGCGTAAATACTTGAACGATATGCGGAAGAAGAATCCAGTCCCTTTTACACAGAAGTTCCCCAGCGCGGATCCCTTGGCACTGCGGCTGTTACAAAGAATGTTAGCATTTGACCCAAAGGATAGGCCAACTGCAAAGGAGGCTTTGGCTGATCCGTACTTCAAGGGCTTGGCTAAGGTGGAGAGAGAACCTTCATGCCTCCCAATCTCAAAAATAGAGTTTGAATTTGAGAGACGAAGGGTAACAAAGGAGGATATCAGGGAGTTAATATACCGTGAGATATTGGAATATCATCCGCAACTGCTCAAAGATTACATGAATGGAAATGAAGGGACAAATTTTATTTATCCTAGTGCTCTTGGTCAGTTCAAGCAGCAGTTTGCACACTTGGAAGAAAACTGTGGTAGGAGTGGCCCGGTTATTCCGCTGGATAGAAAGCATGTTTCACTTCCAAGATCCACAGTACATTGCAGCACCGTACCTCCCAAACCACAACCCATGTCTTTGCGGGATCAACAGCCTAACAGGAACGAAGGCAGGGTTGGTGTAGATGTAAATTCAGGGATGTACTCCTCTGGTGCAAAACAACCACAACCACAACCATCTAGGGTGGTGTCATCCGCAAAACCTGGTAGAGTGGTTGGCCCAGTCTCGTATGAGGTAGGAGACCAAGCCATGAACAACGAAAGCTACAACAACAATCCAGCAGCGAGAATTGTGATGAGAAACGCTGTTGTTCCGCCACAGACCATGTCACTGTCGCAGCAATACTATATGCAAGCTGCAAATGGTGCACCCATGAATCACCAACCCAAGCAGGTTCCAATGAACAGCATGATCACTAACAGACAACCACCTGGGCTCCGGCAGGAAGCCCCAGTCCCAGTTGATAGTAATACTAACATCTACCACCATCCACATCACCAACTTTACCACAAACAACAGCCACAGCCACAGCCCAAGCCTTCTGGTAGTTTTAACAGCAGGGTTGCTCTGGATGCAAAAATAATGCAAGCTCAGTCCCAGTTTGCTGCCACTGCAGTTGCAATGGGTGCTCACAGAAATGTTGCTGGTACTGTTCAGTACGGCATGTCTTAA |
|  |  | Protein | MQQDQRKKSLKDAEFFTDYGDANRYKLLEVIGKGSYGVVCAAIDTHTGEKVAIKKINDIFEHNSDAIRILREVKLLRLLRHPDIVEIKRIMMPPTRRDFKDIYVVFELMESDLHQVIKANDDLTREHHQFFLYQMLRALKYMHTANVYHRDLKPKNILANANCKLKICDFGLARVAFTDAPTTVFWTDYVATRWYRAPELCGSFSSKYTPAIDMWSIGCIFAEVLTGKPLFPGKSVVHQLDLITDLLGKPSQEVISGVRNDKARKYLNDMRKKNPVPFTQKFPSADPLALRLLQRMLAFDPKDRPTAKEALADPYFKGLAKVEREPSCLPISKIEFEFERRRVTKEDIRELIYREILEYHPQLLKDYMNGNEGTNFIYPSALGQFKQQFAHLEENCGRSGPVIPLDRKHVSLPRSTVHCSTVPPKPQPMSLRDQQPNRNEGRVGVDVNSGMYSSGAKQPQPQPSRVVSSAKPGRVVGPVSYEVGDQAMNNESYNNNPAARIVMRNAVVPPQTMSLSQQYYMQAANGAPMNHQPKQVPMNSMITNRQPPGLRQEAPVPVDSNTNIYHHPHHQLYHKQQPQPQPKPSGSFNSRVALDAKIMQAQSQFAATAVAMGAHRNVAGTVQYGMS* |
| LbMAPK18 | Lb0G36729.1 | CDS | ATGGCGACTCCAGTTTCTCCTCCAAACGGGACAGGTCATGAAGGCAAACACTACTTTACAATGTGGCAAACACTGTTCGAGATCGATACAAAATACATCCCCATAAAGCCAATTGGTCGTGGAGCATATGGAACAGTGTGTTCTTCCATTAATAAGGAAAGGAACGAGAAGGTGGCCATAAAGAAGATAAACAATGTTTTTGAGAACCGGATCGATGCTCTGAGAACTCTACGTGAGTTGAAGCTTCTTAGGCATCTGAGGCATGAGAATGTGATTGGTTTGAAGGATGTTATGTTGCCTGTTCAGAGAACGTTCAAGGATGTATATCTCGTGTATGATCTGATGGATACGGATTTGCATCAGATTATAAAGTCTTCCCAGGCTCTGTCGAATGACCATTGCCAGTATTTTTTGTTTCAGTTGCTTCGAGGTCTAAAGTATCTTCACTCAGCCAACATCCTTCACCGAGACCTAAAGCCCGGAAACCTCCTAATCAACGCGAACTGTGACCTGAAGATCTGCGATTTCGGTCTGGCGCGTACAAGCAGCGGCAAGGAAGGGCAGTTCATGACAGAGTACGTAGTCACCCGGTGGTATCGTGCACCAGAGCTTCTCCTGTGCTGTGACAAGTATGACACATCCATCGATGTCTGGTCAGTTGGCTGCATCTTTGCGGAGCTCCTAGGCAGAAAACCAATCTTCCCAGGATCAGAGTGCCTGAACCAGCTGAAGCTCATCATCAACGTCCTTGGAAGCCAGAAGGAGTCAGACATTGAGTTCATTGACAACCCAAAGGCGAAGCGGTTCATCAAGTCACTCCCTTACTCACCAGGCACACCGTTCTCCCGCCTCTACCCGAATGCACACCCCCTGGCACTTGATCTGCTGCAGAGGATGCTGGTTTTTGACCCAACAAAGAGGATTAGTGTGACAGAAGCGCTGCAGCATCCGTACATGTCACCATTGTATGATCCAAGGTCAGACCCACCTGCACAGGTACCGATCAACCTCGATATTGATGAGGAGCTGGATGAGAAGACGATAAGGGAGATGATGTGGATGGAGATCCTGCATTACCATCCGGAAGCTGTTGATGCTGCAGCAGGCGGTGGCATGGATGTCCTCTGCTGA |
|  |  | Protein | MATPVSPPNGTGHEGKHYFTMWQTLFEIDTKYIPIKPIGRGAYGTVCSSINKERNEKVAIKKINNVFENRIDALRTLRELKLLRHLRHENVIGLKDVMLPVQRTFKDVYLVYDLMDTDLHQIIKSSQALSNDHCQYFLFQLLRGLKYLHSANILHRDLKPGNLLINANCDLKICDFGLARTSSGKEGQFMTEYVVTRWYRAPELLLCCDKYDTSIDVWSVGCIFAELLGRKPIFPGSECLNQLKLIINVLGSQKESDIEFIDNPKAKRFIKSLPYSPGTPFSRLYPNAHPLALDLLQRMLVFDPTKRISVTEALQHPYMSPLYDPRSDPPAQVPINLDIDEELDEKTIREMMWMEILHYHPEAVDAAAGGGMDVLC* |
| LbMAPK19 | Lb0G37133.1 | CDS | ATGATTAGTAGCAGTATGCGGTCCTCTTCAAGACGAGATAATCAGTCCATGGTTTGGAGAGGCCGCCGCTACACTGACAATGACACTAACCCCACACCTTCAGAGCTAGGGATGATGATGCAGGAGCTTTCTATCAAGAACCTGCAATCTGATGCAGCCACAAAGGCTCGTTACTATATAGCGGGTACACTGTTTGAAGTGGATGCAAAGTATGCTCCAGTTAAGTTACTCGGTAGTGGAGCTTATGGGACAGTCTGCTCTTTCATTAACATACAAACTGGAGAAATTGTTGCTGTCAAGAAGGTTAGAGATGCATTCTTTTGTGACGAAGCTGGTATAAGAACCCTAAGGGAGATAAGAATACTGAAGCATTTAAATCATCCAAACGTGATTAGTTTGAAGGATGTGATGCTCCCAATTGAGAGGTTCAGGTTTAATGAAACGTATCTTGTTTATGAGCTTATGAATGGTGATCTTAATGAGGTTATCAGGTCGGGTTTTCCACTCTCTCAGGATCGATGCAGACACTTTGTTCTCCAGATACTGAAAGGGCTCAAGTACCTACATAACGGGAACGTCCTCCACCGTGATTTGAAACCAGAAAACATCTTCATTGACAAAAACCATAACATCAAGATTGGCGACTTTGGTCTTGCAAGAACAACAACAACTGAATATCTAGGTCAAGACGCAAACTGGCATTTCACAAACTACGTCGCTTCTAGATGGTACCGTGCACCAGAGCTTCTTCTCGGTTGTCCTTACGGTAAAGCTATTGATATGTGGTCAGTTGGTTGCATCTTCGCTGAGCTTCTAGCGGGTAAACCTCTCTTACCAGGATATGACAAAATGAACCAGCTTGACCTTATTCTCTTGCTTCTTGGAAGACAAGAGGAGTCTGATGTTGATTTTGTGAGCTCTGAGAAGTGGAAGGAATATCTACTGAAGCGTTCAAAGGGTGTAATAATGTCTGGAATTGATATGTTCAAGTTATTCCCAAAGGCAGAGAGGCTAGCTGTTGATCTATTGAGGAGGATGCTTGTTTTTAACCCCAAGGATAGAATCAGTGTTGCAGAGGCGTTACATCATCCTTATTTTTGTGAGATTTATAATCCTATGCTGGATGTACCGGACTTGGATCCTATTGATGTGGAACTCAAGGAGGGTGAGGTGGTTGATTTTAGAGAGTTAGTTTGGAAGGAAATGCTAGCTTATTAA |
|  |  | Protein | MISSSMRSSSRRDNQSMVWRGRRYTDNDTNPTPSELGMMMQELSIKNLQSDAATKARYYIAGTLFEVDAKYAPVKLLGSGAYGTVCSFINIQTGEIVAVKKVRDAFFCDEAGIRTLREIRILKHLNHPNVISLKDVMLPIERFRFNETYLVYELMNGDLNEVIRSGFPLSQDRCRHFVLQILKGLKYLHNGNVLHRDLKPENIFIDKNHNIKIGDFGLARTTTTEYLGQDANWHFTNYVASRWYRAPELLLGCPYGKAIDMWSVGCIFAELLAGKPLLPGYDKMNQLDLILLLLGRQEESDVDFVSSEKWKEYLLKRSKGVIMSGIDMFKLFPKAERLAVDLLRRMLVFNPKDRISVAEALHHPYFCEIYNPMLDVPDLDPIDVELKEGEVVDFRELVWKEMLAY* |
| LbMAPK20 | Lb0G37186.1 | CDS | ATGGACCATGAAAATGTGGTTGCAATCAGAGATATTATACCTCCTCCGAGAAGAGAGGCTTTCAATGATGTGTATATTGCATATGAACTAATGGACACAGATCTTCATCAAATTATCCGCTCCAATCAAGCTTTATCAGAGGAGCATTGCCAGTATTTTCTCTACCAAATTCTTCGTGGATTGAAGTACATACACTCAGCAAATGTTCTCCACAGGGACTTGAAGCCCAGCAACTTGCTACTAAATGCTAATTGTGATCTAAAAATATGTGATTTTGGACTTGCCCGTGTGACATCCGAAACTGATTTTATGACTGAATATGTTGTTACGAGATGGTATCGTGCACCAGAGCTGTTGTTGAACTCTTCAGATTACACTGCAGCTATTGACATATGGTCTGTTGGCTGCATATTTATGGAACTAATGGATCGCAAGCCTCTGTTCCCTGGTAGGGATCATGTTCATCAGCTACGACTACTTATGGAGCTGATTGGCACTCCTTCAGAGGCTGAGTTGGGGTTTTTAAATGATAATGCTAAAAGATATATTCGGCAACTTCCTATGCACAGAAGGCAGTCGCTCCTGGAAAAGTTCCCACATGTGCACCCATCAGCCATTGATCTTATTGAGAAGATGTTGACGTTTGATCCTAGGCAACGAATAACAGTTGAAGACGCGCTTGCACATCCATACCTGAACTCATTGCATGATATAAGTGATGAGCCAGTATGCACGACTCCATTCAACTTTGATTTCGAGCAGCATGCTTTGACAGAGGAGCAGATGAGGGAGCTCATATACCGGGAGGCACTTGCTTTCAACCCTGAGTATCAACAGTGA |
|  |  | Protein | MDHENVVAIRDIIPPPRREAFNDVYIAYELMDTDLHQIIRSNQALSEEHCQYFLYQILRGLKYIHSANVLHRDLKPSNLLLNANCDLKICDFGLARVTSETDFMTEYVVTRWYRAPELLLNSSDYTAAIDIWSVGCIFMELMDRKPLFPGRDHVHQLRLLMELIGTPSEAELGFLNDNAKRYIRQLPMHRRQSLLEKFPHVHPSAIDLIEKMLTFDPRQRITVEDALAHPYLNSLHDISDEPVCTTPFNFDFEQHALTEEQMRELIYREALAFNPEYQQ* |
