# Supplementary material for: Genome-wide identification of the mitogen-activated kinase gene family from Limonium bicolor and functional characterization of LbMAPK2 under salt stress
Source: BMC Plant Biol. 2023 Nov 15;23:565. doi: 10.1186/s12870-023-04589-x (PMC10647163; doi:10.1186/s12870-023-04589-x)
Supplement: Supplementary file 8 — Additional file 8: Figure S5. The expression level of different lines of LbMAPK10. [file 12870_2023_4589_MOESM8_ESM.docx]

**Table S2** **The primers for 20 *MAPK* genes used in real-time qPCR analysis**

| **Gene ID** | **F1 (5'**-**3')** | **F2 (5'**-**3')** |
| --- | --- | --- |
| *qLbMAPK1* | CTTCGGCTTCTTCACCAT | ACTTGATGTAGGTCTGATTCC |
| *qLbMAPK2* | GACTCATCAATACGCTTCT | GTTGCTTCACATAGTTCTTC |
| *qLbMAPK3* | CTCCTGCTGAATGCTAAT | CACAACATACTCGGTCAT |
| *qLbMAPK4* | CTCCTGCTGAATGCTAAT | CACAACATACTCGGTCAT |
| *qLbMAPK5* | CTCCTGCTGAATGCTAAT | CACAACATACTCGGTCAT |
| *qLbMAPK6* | CTCCTGCTGAATGCTAAT | CACAACATACTCGGTCAT |
| *qLbMAPK7* | CTGGCAAGGATTATGTTCATCA | AACCGAGGCTGGATTCAT |
| *qLbMAPK8* | ATGGTGAAGCAAGTAGGTAT | CAATGGCAGAAGCAACAA |
| *qLbMAPK9* | CGTCAACTCTTCTTCTTC | CCACTCTCATAATCTACAAC |
| *qLbMAPK10* | GTTGCTTAGGCTATTGAG | CTTGATGACTTGGTGAAG |
| *qLbMAPK11* | TATTCTGGACGGACTATGTT | ATGTCTATCGCTGGTGTAT |
| *qLbMAPK12* | AAGCCAGCCAATCTATTC | CCACCTTGAGACAACATAA |
| *qLbMAPK13* | CTATGAATGACGAGAAGACT | ATACACCAACTTGCTCAG |
| *qLbMAPK14* | TATTCTGGACGGACTATGTT | ATGTCTATCGCTGGTGTAT |
| *qLbMAPK15* | AGTCGTTATCTCATCCTTAC | TCATCTCTTCCTCTCCAA |
| *qLbMAPK16* | ACATCCATACCTGAACTCAT | CATCTGCTCCTCTGTCAA |
| *qLbMAPK17* | GGAGTTATGGAGTTGTCT | GGATAGCATCAGAGTTGT |
| *qLbMAPK18* | TATCTTCACTCAGCCAAC | ATCTTCAGGTCACAGTTC |
| *qLbMAPK19* | GACCTTATTCTCTTGCTTCT | CGCTTCAGTAGATATTCCTT |
| *qLbMAPK20* | ACATCCATACCTGAACTCAT | CATCTGCTCCTCTGTCAA |
