# Supplementary material for: Genome-wide identification of the mitogen-activated kinase gene family from Limonium bicolor and functional characterization of LbMAPK2 under salt stress
Source: BMC Plant Biol. 2023 Nov 15;23:565. doi: 10.1186/s12870-023-04589-x (PMC10647163; doi:10.1186/s12870-023-04589-x)
Supplement: Supplementary file 9 — Supplementary Material 9 [file 12870_2023_4589_MOESM9_ESM.docx]

**Table S3** **Primers of genes associated with salt gland development**

| **Gene ID** | **F1 (5'**-**3')** | **F2 (5'**-**3')** |
| --- | --- | --- |
| *LbTTG1* | GACGACGAATCTCTTACC | TTGGCTGGAACATTAACT |
| *LbCPC* | GAGACTACAATTAGGAGAAGGC | TATCCGACCTGCTATCAA |
| *LbEGL3* | AGACATCAAGACGAGGAA | TCAACTGCTCACTTCTC |
| *LbGL2* | TTCATCCTCGTCAAGTCAA | AGTTCTCATGGCGTTCTT |
